# Docirbrutinib is a pan-mutant BTK inhibitor and inhibits B-cell receptor signaling in chronic lymphocytic leukemia cells in preclinical and early clinical investigations

**DOI:** 10.1038/s41408-026-01509-8

**Published:** 2026-05-07

**Authors:** Natalia Timofeeva, Breana Herrera, Hitomi Fujiwara, Tokiko Asami, Hiroko Endo, Mariko Hatakeyama, Fumio Nakajima, Hiroshi Ohmoto, Yu Nishioka, Kyoko Miyamoto, Akinori Arimura, Shady I. Tantawy, Javier Pinilla-Ibarz, Catherine C. Coombs, Nitin Jain, Masaaki Sawa, Varsha Gandhi

**Affiliations:** 1 Department of Translational Molecular Pathology, The University of Texas MD Anderson Cancer Center, Houston, TX USA; 2 Carna Biosciences, Inc., Kobe, Japan; 3 CarnaBio USA Inc., South San Francisco, CA USA; 4 Internal Medicine and Clinical Hematology Department, Suez Canal University, Ismailia, Egypt; 5 Moffitt Cancer Center, Tampa, FL USA; 6 University of California, Irvine, CA USA; 7 Department of Leukemia, The University of Texas MD Anderson Cancer Center, Houston, TX USA

**Keywords:** Cancer therapeutic resistance, Drug development

## Abstract

BTK inhibitors (BTKi) have become standard of care for treatment of patients with chronic lymphocytic leukemia (CLL). Covalent BTKi (cBTKi) such as ibrutinib, acalabrutinib, and zanubrutinib are effective but alterations in the kinase domain at C481 or BTK gatekeeper residue T474 mutations result in development of resistance. Noncovalent BTK inhibitors (ncBTKi) such as pirtobrutinib are effective in patients with C481x mutations developed through use of cBTKi. However, resistance to ncBTKi can occur owing to second site aberrations in *BTK,* generating novel mutations such as L528x and T316x. Sometimes, CLL cells with double BTK mutations are also observed. These BTK aberrations underscore a need for new inhibitors that target pan-BTK-mutants. We evaluated the efficacy of a new ncBTKi, docirbrutinib (AS-1763), against 14 BTK mutants, including C481S, T474x, and L528x, as well as gatekeeper and kinase domain double mutants, using biochemical assays, cell-line models, and primary CLL lymphocytes. Docirbrutinib potently inhibited BTK autophosphorylation and mutant BTK–driven cell proliferation, with greater effects than ibrutinib and pirtobrutinib against certain mutants. In treatment-naïve and relapsed/refractory CLL samples, docirbrutinib disrupted B-cell receptor signaling and sensitized cells to apoptosis induced by venetoclax and AZD5991. In a dose-escalation trial (NCT05602363), docirbrutinib decreased CCL3/CCL4 biomarkers and inhibited the B-cell receptor pathway signaling in longitudinal samples from patients with relapsed/refractory CLL. These findings establish docirbrutinib as a pan-mutant ncBTKi with potential to improve outcomes for CLL patients, including those with disease resistant to cBTKi and other ncBTKi.

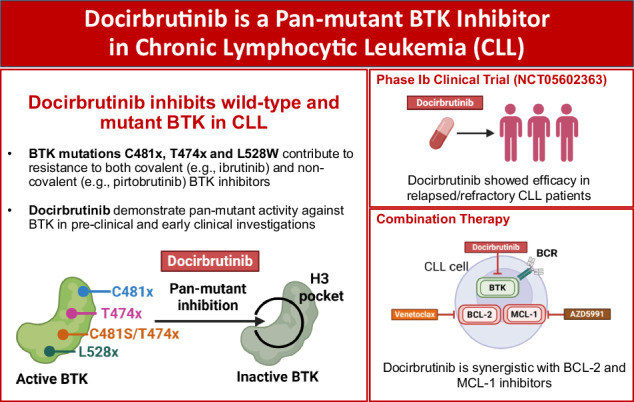

## Introduction

Chronic lymphocytic leukemia (CLL) is characterized by the expansion of clonal B-cells [1]. CLL pathophysiology involves two main mechanisms: aberrant B-cell receptor (BCR) signaling and dysregulation of anti-apoptotic proteins. Bruton’s tyrosine kinase (BTK), a key enzyme in the BCR pathway, is thus a valuable therapeutic target [2], and BTK inhibitors (BTKi) have emerged as a cornerstone of CLL treatment [2, 3]. Additionally, overexpression of anti-apoptotic proteins such as BCL-2 and MCL-1 contributes to CLL cell survival, prompting exploration of BCL-2 and MCL-1 inhibitors [4].

Covalent BTKi (cBTKi), such as ibrutinib [2], acalabrutinib [5], and zanubrutinib [6], irreversibly bind to BTK by forming a covalent bond with cysteine 481 (Cys481), thereby inhibiting its kinase activity. Despite their clinical success, resistance often develops due to Cys481 mutations, rendering cBTKi ineffective [7]. To address this, noncovalent BTKi (ncBTKi) were developed to inhibit both wild-type (WT) and mutated BTK. The ncBTKi, represented by pirtobrutinib, reversibly binds to the ATP binding site of BTK without forming a covalent bond with C481, thereby retaining activity against C481 resistant mutations [8–10]. Pirtobrutinib has demonstrated high response rates in relapsed/refractory CLL, including patients who failed prior cBTKi therapy [10, 11]. Nevertheless, resistance mutations outside Cys481 in the BTK kinase domain have emerged, reducing its efficacy [12]. Published studies distinguish two functional classes. Mutations at the gatekeeper BTK residue T474 (e.g. T474I, T474F) alter access to the ATP-binding site and the regulatory spine of BTK, but maintain BTK catalytic activity, conferring resistance to both cBTKi and ncBTKi [13, 14]. In contrast, kinase-impaired or kinase-dead variants (e.g., C481R, C481F, L528W, and V416L [15]), disrupt BTK’s catalytic function while paradoxically enabling BCR signaling through noncatalytic scaffolding, allowing CLL cells to bypass BTK inhibition [12, 16]. Moreover, the efficacy of BTKi can also be undermined by downstream mutations (e.g., PLCγ2) or alterations in non-BTK pathways activating BCR signaling [17]. There is a need for next-generation ncBTKi capable of inhibiting a broader spectrum of mutations, particularly mutations at the gatekeeper residue T474 in BTK and kinase-dead mutations such as L528W.

Venetoclax, a potent and selective BCL-2 inhibitor, has demonstrated remarkable efficacy in CLL, both as a monotherapy and in combination with ibrutinib in preclinical [18–20] and clinical settings [21–25]. It binds the BH3 pocket of BCL-2, inducing apoptosis [26]. However, *BCL2* mutations such as G101V induce conformational changes in the BH3 binding pocket, significantly reducing the efficacy of venetoclax [27].

MCL-1, another anti-apoptotic target overexpressed in CLL [28, 29] It promotes survival by binding BIM/BAK and preventing mitochondrial outer membrane permeabilization [30]. The efficacy of MCL-1 inhibitors was demonstrated in preclinical trials [29, 31]. However, MCL-1 inhibitors have been associated with cardiotoxicity [32, 33] and efforts are ongoing to mitigate the risk.

BTKi and BCL-2 inhibitors offer complementary mechanisms and distinct toxicity profiles, making them suitable for combination therapy [12, 34] that can be beneficial for both first-line [21, 22], as well as relapsed CLL [23, 35]. These agents are also used sequentially in practice, and resistance to venetoclax often involves the BCL-2 G101V mutation. We therefore include a brief overview of BCL-2 mutations to place our BTK-focused analyses in clinical context and to motivate sequencing and combination strategies.

To address resistance limitations, we developed a novel ncBTKi, docirbrutinib (AS-1763). We previously reported the synthesis, chemical structure, kinase selectivity, and in vivo antitumor activity of docirbrutinib in BTK WT and C481S models [36]. Here, we extend our research and investigate docirbrutinib’s effectiveness using enzymatic assays, variety of BTK mutant cell lines and primary CLL samples (naïve and relapsed/refractory), and samples from a phase Ib clinical trial (NCT05602363) of docirbrutinib in patients with previously treated CLL [37].

## Materials, methods, and patients

### Drugs

Docirbrutinib was obtained from Carna Biosciences; ibrutinib, acalabrutinib, pirtobrutinib, venetoclax, and AZD5991 were purchased from MedChemExpress.

### In vitro kinase assay

The inhibitory potency of BTKi against BTK was evaluated using the ADP-Glo Max assay kit (Promega); however, for BTK-inactive mutants, LanthaScreen Eu kinase binding assay kit (Thermo Fisher Scientific) was used. Details are in Supplemental Methods.

### Rapid dilution assay

BTK activity was measured using the MSA assay kit (QuickScout Screening Assist Kit, Carna Biosciences, Inc.) [36]. The off-rate of docirbrutinib was measured using the rapid dilution assay, which was performed according to the procedure described [38].

### Cell lines

HEK293 cells (RRID:CVCL_0045) were purchased from ATCC (Manassas, VA, USA). OCI-Ly10 cells (RRID:CVCL_8795) were obtained from University Health Network, Ontario, Canada. Ramos cell line (RRID:CVCL_1646) was purchased from ATCC (Manassas, VA, USA). All cell lines were tested negative for mycoplasma contamination. Details are in Supplemental Methods.

### pBTK inhibition assay in HEK293 cells

Cellular potency of BTKi was assessed by measuring the phosphorylation levels at the autophosphorylation site Tyr223 of BTK in HEK293 cells expressing the corresponding *BTK* mutations introduced by either plasmid transfection or retroviral transduction. Details are in Supplemental Methods.

### Resazurin assay in OCI-Ly10 cells

Antiproliferative potencies of compounds were measured using resazurin assay. OCI-Ly10 cells harboring WT or mutant BTK were treated with BTKi for 96 h, and then cell viability was measured [36].

### Isobologram analysis

Synergy between docirbrutinib, venetoclax, and AZD5991 in OCI-Ly10 cells was assessed by the isobologram analysis [39]. Briefly, DMSO stock solutions (10,000-fold of each IC_50_) of each compound were prepared and mixed at fixed ratios. Cells were treated for 96 h, and then cell viability was determined by resazurin assay. IC₅₀ values were determined for each compound alone and for the fixed-ratio combinations. Fractional inhibitory concentrations (FIC) were calculated for each combination point, and isobolograms were constructed to evaluate synergy.

### Apoptosis assay in OCI-Ly10 cells

The apoptosis assay was carried out in OCI-Ly10 cells harboring mutant BTK using a commercial assay kit (FITC Annexin V Apoptosis Detection Kit with 7-AAD, BioLegend, or Guava Nexin Reagent, Millipore) according to the manufacturer’s protocol. Data were acquired with a Sony SA3800 spectral cell analyzer and analyzed using FlowJo (RRID:SCR_008520) software.

### Modeling study

Molecular modeling was performed using the suite of programs within Biovia Discovery Studio 2024 (DASSAULT SYSTEMS). Docirbrutinib was flexibly docked into the active site of BTK (5ZZ4) using Flexible Docking Module with a standard parameter setup. Homology models of BTK L528W were built from structural data of BTK (5ZZ4).

### Patients

Blood samples were obtained from 30 treatment-naïve, 22 relapsed/refractory CLL patients, and patients from docirbrutinib clinical trial (NCT05602363). Three men and 1 woman with median age 75.5 (62–78) years were enrolled at the MD Anderson Cancer Center (MD Anderson). All patients provided written informed consent to participate in the clinical trial and laboratory correlative studies. The sample collection protocol and the clinical trial were approved by the Institutional Review Board of MD Anderson in accordance with the Declaration of Helsinki. The inclusion and exclusion criteria are provided in the study information at clinicaltrials.gov. Clinical characteristics of CLL patients are listed in Supplemental Tables S1 and S2. Sample size was determined by the availability of primary patient material and enrollment at MD Anderson Cancer Center, which is typical for translational ex vivo studies using primary CLL samples [9, 40, 41]. The cohort size is consistent with prior published studies evaluating ex vivo drug sensitivity and was sufficient to detect biologically meaningful differences in cell viability across treatment conditions. For the docirbrutinib clinical trial correlative studies, only 4 patients were enrolled at our site. To maximize inferential efficiency despite small enrollment, we used a repeated-measures design with paired baseline and multiple on-treatment time points. Peripheral blood samples were collected from patients before docirbrutinib therapy, on cycle 1 day 1 (C1D1), and at multiple time-points: 1 week (cycle 1 day 8), 4 weeks (cycle 2 day 1), and 8 weeks (cycle 3 day 1).

### Processing of blood samples from patients

Blood samples were diluted with PBS (Corning), and mononuclear cells were isolated using the Ficoll-Hypaque density centrifugation method. Isolated mononuclear cells were considered primary CLL cells.

### Analyses of primary CLL cells and samples from the docirbrutinib clinical trial

#### Cell migration, proliferation, apoptosis, calcium release, MitoSOX and B-cell activation assays

Cell migration (transwell), proliferation (resazurin-based), apoptosis (Annexin V/PI), calcium release (Rhod-2 AM), and B-cell activation (CD86 expression) assays procedures were standard and described in detail in Supplemental Methods.

### CCL3 and CCL4 ELISA

Plasma samples were tested for CCL3 and CCL4 levels using Quantikine ELISA kit (R&D Systems).

### Immunoblot assay (primary CLL cells)

Primary CLL cells were lysed in radio-immunoprecipitation assay buffer (Millipore-Sigma) supplemented with complete mini protease inhibitor tablets (Roche). Protein concentrations were determined using the Pierce BCA Protein Assay Kit (Thermo Fisher Scientific). Immunoblot assays were performed as described in the Supplemental Methods. Primary antibodies are listed in Supplemental Table S3.

### Next-generation sequencing of clinical samples

We performed next-generation sequencing of 162 genes (see Supplemental Methods) associated with B-cell neoplasms (Supplemental Table S4).

### Pharmacokinetic analysis of clinical samples

Plasma concentrations of docirbrutinib were determined by Worldwide Clinical Trials Early Phase Services/Bioanalytical Sciences, LLC (Austin, Texas) using a validated liquid chromatography-tandem mass spectrometry method.

### Statistical analysis

Data are expressed as mean ± the standard error of the mean. For primary samples, individual data points are shown in all graphs. Data distribution within each group was assessed using the Shapiro–Wilk test and visual inspection of Q–Q plots. For data that did not meet normality assumptions, the Wilcoxon test was used for group comparisons. For normally distributed data, paired two-tailed Student’s t-tests were used to compare two groups. A *P* value < 0.05 was considered statistically significant. Graphs and t-tests were generated using GraphPad Prism (RRID:SCR_000306) v10.3.1. For OCI-Ly10 apoptosis assays, significance was evaluated using a one-way ANOVA followed by Sidak’s multiple comparison test. Drug interaction analyses in combination studies were performed using CompuSyn (RRID:SCR_022931) based on the Chou-Talalay method [39].

## Results

### Docirbrutinib is a pan-mutant ncBTKi with a slow off-rate

**Fig. 1:**
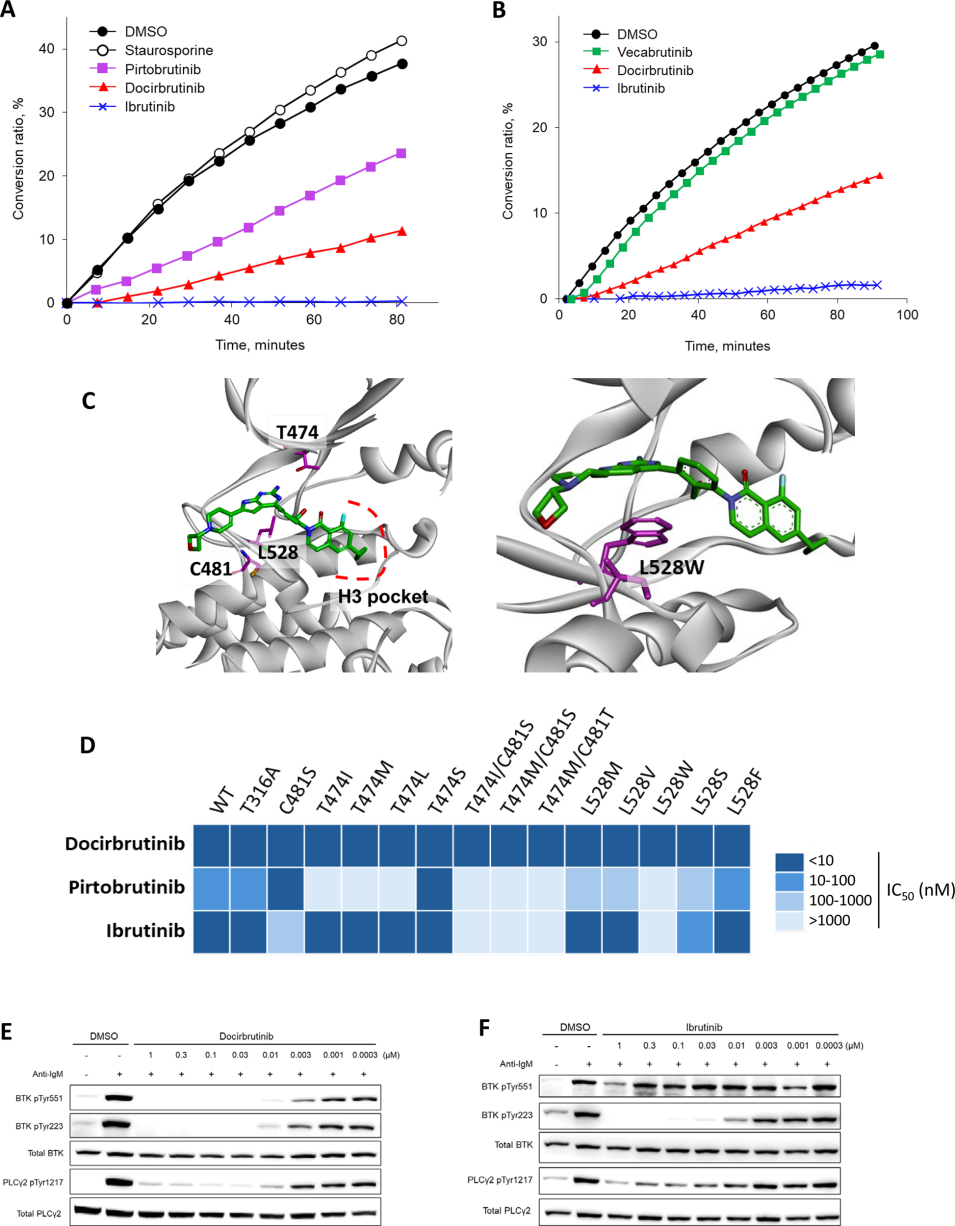
Docirbrutinib, a highly potent ncBTKi with slow off-rate, inhibits BCR signaling and blocks calcium release in Ramos cells. **A**, **B** Rapid dilution assay for evaluation of the off-rate. The enzymatic reaction was initiated by diluting a pre-incubated enzyme-inhibitor mixture into assay buffer containing substrates. Recovery of enzymatic activity was monitored by formation of the phosphorylated product, which reflects the dissociation of inhibitor from the enzyme. **C** Docking study of Docirbrutinib. Docirbrutinib can bind to BTK without steric repulsion on T474 and L528 mutations. Left: Docking study of docirbrutinib bound to an inactive conformation of WT BTK (PDB ID: 5ZZ4). Right: Docking study of docirbrutinib bound to BTK L528W mutant. **D** Heat map of inhibitory potency of BTK inhibitors for BTK mutants. Docirbrutinib demonstrated pan-mutant BTK inhibition at low nanomolar range in recombinant enzymatic assays. **E**, **F** Dose-dependent inhibition of BTK and PLCγ2 phosphorylation by docirbrutinib or ibrutinib. Ramos cells were treated with docirbrutinib and ibrutinib for 1 h and then stimulated with anti-IgM for 10 min. The phosphorylation levels of BTK (Tyr223 and Tyr551) and PLCγ2 (Tyr1217) were analyzed by Western blotting. Ibrutinib inhibited only the phosphorylation of Tyr223 and failed to inhibit phosphorylation at Tyr551.

Docirbrutinib was originally identified as a potent, selective, noncovalent inhibitor of both WT and C481S-mutant BTK. It exhibited potent antitumor activity in xenograft mouse models implanted with ibrutinib-resistant BTK C481S mutant diffuse large B-cell lymphoma (DLBCL) cells [36]. A docking study of docirbrutinib bound to WT BTK suggested that it interacts with an inactive conformation of BTK, in which the cyclopropyl-isoquinolinone group of docirbrutinib occupied a back pocket of BTK, called the H3 pocket, which is reported to form in the unactivated state of BTK [42]. Since inhibitors that bind to an inactive conformation of a kinase were previously reported to have slow off-rates, so we evaluated the off-rates of docirbrutinib by a rapid dilution assay. As shown in Fig. 1A, the product formation rate after staurosporine treatment was indistinguishable from that after treatment with DMSO, and no product formation was observed after treatment with the cBTKi ibrutinib. In contrast, ncBTKi treatments allowed to produce the product, but the product formation rate after docirbrutinib treatment was very slow compared with that of pirtobrutinib. Vecabrutinib, another ncBTKi, showed no difference in product formation compared to the vehicle control (Fig. 1B). The docking study also suggested that docirbrutinib can bind to BTK without steric clashes caused by T474 and L528 mutations (Fig. 1C), prompting us to further characterize its inhibitory profile against other resistant mutants. To this end, we produced recombinant BTK mutant proteins, either documented in the literature or predicted based on single-nucleotide codon changes. Kinase activity was evaluated using the ADP-Glo^TM^ kinase assay. As both A428D and L528W are recognized as kinase-dead mutations, we employed the LanthaScreen^TM^ Eu kinase binding assay to assess their binding properties. While the assay was successfully established for the L528W mutant, it failed for the A428D mutant due to technical limitations. Therefore, 14 mutants were included in the analysis presented in this study (Fig. 1D**;** Supplemental Tables S5-S7). Profiling of docirbrutinib using the panel of BTK mutants revealed that BTK mutants resistant to ibrutinib or pirtobrutinib were sensitive to docirbrutinib with sensitivity similar to that of WT and C481S mutants, indicating that docirbrutinib is a potent BTKi with a broad spectrum of activity against resistant mutants (Table 1). We next evaluated the effects on anti-IgM-stimulated BCR signaling in Ramos cells. The enzymatic activity of BTK is regulated by upstream kinases (SYK and Lyn), which phosphorylate Tyr551 to induce a conformational change upon BCR activation [43]. Compounds interacting with the H3 pocket were reported to bind a Src-like inactive conformation of BTK, sequestering Tyr551 from the upstream kinases and allowing Tyr551 to remain unphosphorylated [42]. Docirbrutinib potently inhibited phosphorylation of Tyr551 in addition to Tyr223, an autophosphorylation site (Fig. 1E), suggesting that docirbrutinib binds a Src-like inactive conformation of BTK, as predicted by our docking model study. In contrast, ibrutinib inhibited only the phosphorylation of Tyr223 and failed to inhibit phosphorylation at Tyr551, supporting that the binding mode of docirbrutinib differs from that of ibrutinib (Fig. 1F).

**Table 1:** IC_50_ values BTKi against BTK mutants.

BTK mutant	IC_50,_ nM^a^		
	Docirbrutinib	Pirtobrutinib	Ibrutinib
WT	0.9 ± 0.1	11.2 ± 2.3	0.7 ± 0.1
T316A	1.3 ± 0.1	41.4 ± 3.0	0.6 ± 0.0
C481S	0.8 ± 0.1	8.5 ± 2.4	178 ± 12
T474I	1.5 ± 0.2	> 8016	1.4 ± 0.3
T474M	0.8 ± 0.1	> 10000	3.8 ± 0.5
T474L	2.2 ± 0.8	> 10000	7.3 ± 0.9
T474S	1.5 ± 0.1	5.4 ± 1.4	0.9 ± 0.0
T474I/C481S	1.4 ± 0.2	7049 ± 616	> 10000
T474M/C481S	0.9 ± 0.1	> 10000	> 10000
T474M/C481T	0.8 ± 0.1	> 10000	> 10000
L528M	1.1 ± 0.1	152 ± 22	0.8 ± 0.1
L528V	0.7 ± 0.1	871 ± 47	0.7 ± 0.1
L528W^b^	1.5 ± 0.1	> 10000	2406 ± 466
L528S^b^	3.2 ± 0.3	272 ± 35	15.6 ± 1.6
L528F^b^	1.6 ± 0.1	12.0 ± 0.4	1.6 ± 0.2

^a^IC_50_ values were determined in the presence of 1 mM ATP by ADP-Glo assay.

^b^IC_50_ values were determined by LanthaScreen Eu kinase binding assay.

### Docirbrutinib inhibits multiple BTK mutants in cellular models

**Fig. 2:**
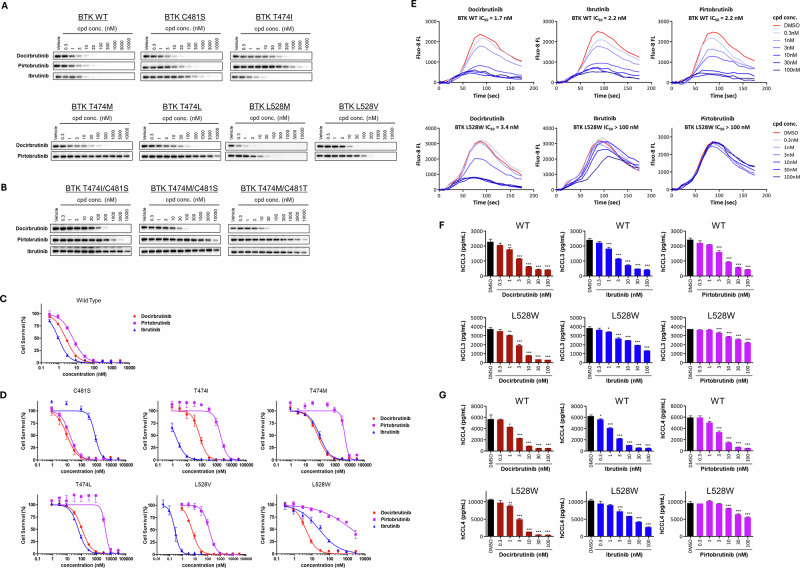
Docirbrutinib’s effect on BTK phosphorylation and on proliferation in cell lines with mutated BTK; comparison with other BTKi. **A**, **B** Docirbrutinib inhibited autophosphorylation of BTK in HEK293 cells transfected with single- (**A**) or double-mutant (**B**) BTK. BTK mutants were transfected into HEK293 cells, and the cells were treated with BTKi for 24 h. Then the phosphorylation levels of pTyr223 were analyzed using Western blotting. conc., concentration. **C**, **D** Antiproliferative activity of BTKi against OCI-Ly10 cells with BTK wild type (**C**) and harboring resistant BTK mutations (**D**). The cells were treated with BTKi for 96 h, and then cell viability was determined by resazurin assay. Data are expressed as the mean ± the standard deviation. **E** Dose-dependent inhibition of BCR pathway by docirbrutinib. Inhibition of calcium flux in OCI-Ly10 cells overexpressing BTK WT or BTK L528W following anti-IgM stimulation in the presence of indicated concentrations of BTK inhibitors. **F**, **G** Docirbrutinib inhibits chemokine secretion in BTK WT and L528W mutant cells. OCI-Ly10 cells overexpressing BTK WT or BTK L528W were treated with BTK inhibitors for 19 h. Levels of secreted chemokines CCL3 (**F**) and CCL4 (**G**) were quantified by ELISA prior to the onset of measurable cell death. The significance of results was evaluated using a Dunnet test. *p < 0.05, **p < 0.01, ***p < 0.001, **** *p* < 0.0001.

To specifically evaluate how BTK mutations affect sensitivity to docirbrutinib and pirtobrutinib without interference from endogenous BCR signaling components, we employed a reconstituted HEK293 cell system that is routinely used to test BTKi resistance. [14, 16, 44, 45] Because HEK293 cells lack endogenous expression of both BCR and BTK, this model provides a “null background” that enables precise assessment of mutant BTK–driven autophosphorylation and its inhibition by BTK inhibitors in a cellular setting. As shown in Fig. 2A, docirbrutinib strongly inhibited wild type, single-mutant BTKs in cells, with nanomolar IC_50_ values (Supplemental Table S8). Pirtobrutinib had substantially lower inhibitory activity than docirbrutinib against T474 and L528 mutants. For T474/C481 double mutants, ibrutinib completely lost inhibitory activity (IC_50_ > 10,000 nM) (Fig. 2B, Supplemental Table S8), while docirbrutinib retained inhibitory activity against these double mutants.

To further assess the therapeutic potential of docirbrutinib, we next evaluated its antiproliferative activity in OCI-Ly10 cells, a human ABC-subtype DLBCL cell line. Docirbrutinib exhibited strong antiproliferative activity against OCI-Ly10 cells, with an IC_50_ value of 2.5 nM, which was comparable to the IC_50_ values of ibrutinib and pirtobrutinib (1.0 and 6.4 nM, respectively) (Fig. 2C). Further, we generated OCI-Ly10 cells harboring the resistant mutations by CRISPR/Cas9-mediated gene editing to examine cytotoxicity. Docirbrutinib exhibited strong antiproliferative activity across all mutants tested. Pirtobrutinib was effective against the C481S mutant but showed limited activity against the other mutants, consistent with the results observed in HEK293 cells. For L528W kinase dead mutant, we generated OCI-Ly10 cells overexpressing BTK L528W to hijack BCR signaling. Overexpression of L528W mutant in OCI-Ly10 cells conferred resistance to ibrutinib and pirtobrutinib, but docirbrutinib maintained the antiproliferative activity against the L528W mutant cells (Fig. 2D, Supplemental Table S9). To further validate the efficacy on L528W mutant, Ca^2+^ flux in OCI-Ly10 cells stimulated with anti-IgM in the presence of indicated BTKi concentrations was measured (Fig. 2E). BCR signaling remains active in kinase-dead BTK L528W mutant cells upon anti-IgM stimulation, suggesting that BTK may function as a scaffold protein within the signaling pathway. Notably, docirbrutinib effectively blocked BCR signaling in both BTK WT and L528W mutant cells with comparable potency, whereas ibrutinib and pirtobrutinib showed inhibitory effects only in BTK WT cells. We also confirmed that docirbrutinib suppressed the secretion of chemokines CCL3 and CCL4 in both BTK WT and L528W mutant cells, while ibrutinib and pirtobrutinib failed to inhibit chemokine production in the L528W mutant cells (Fig. 2F&G).

### Docirbrutinib in primary treatment naïve and previously treated CLL cells

**Fig. 3:**
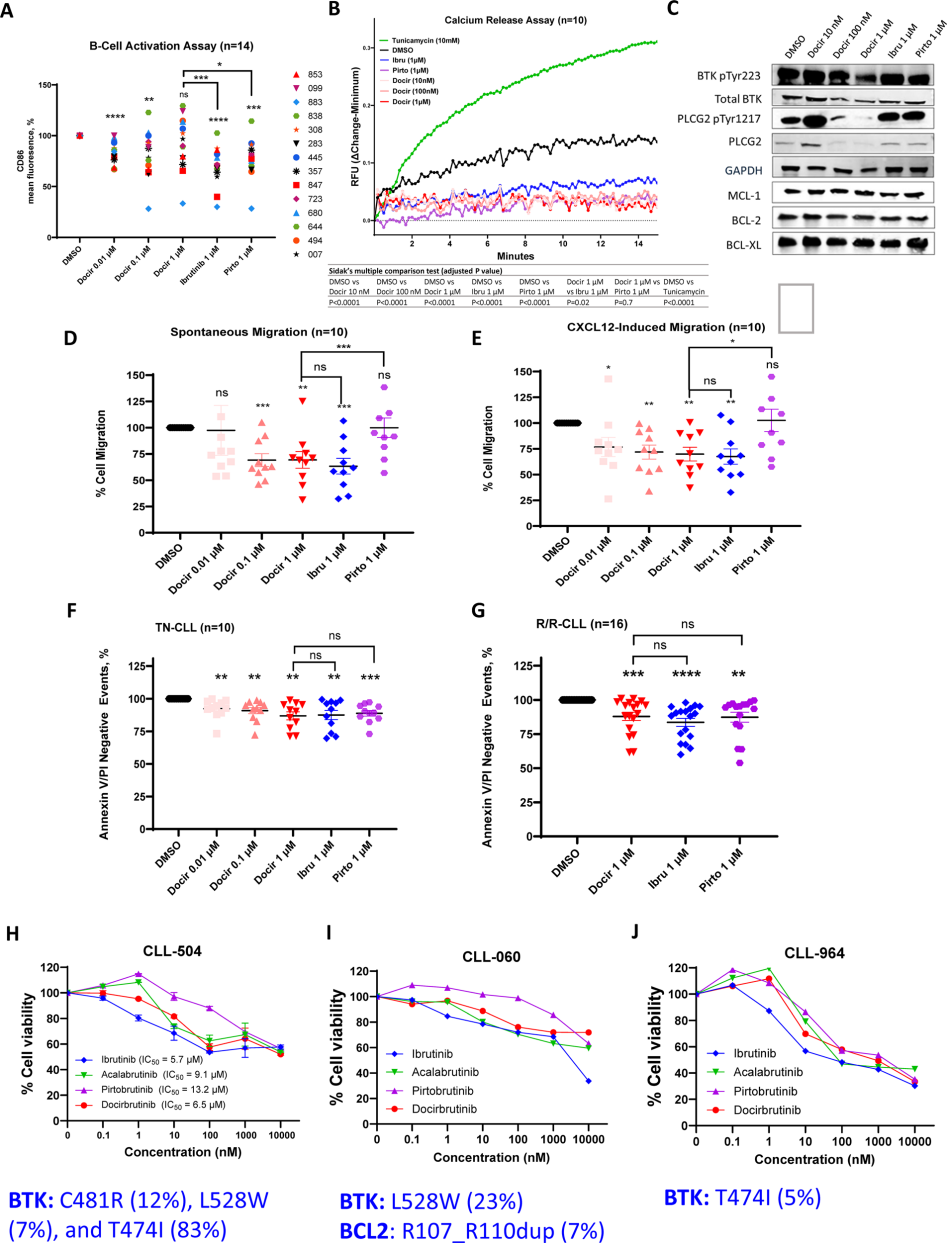
Comparison of docirbrutinib to pirtobrutinib and ibrutinib in CLL lymphocytes. **A**. Inhibition of B-cell activation by BTKi. Fourteen primary CLL samples were treated for 24 h and stained with CD86 for analysis. Each symbol represent an individual patient’s sample. **B**. Inhibiton of calcium release by BTKi. Primary CLL cells were treated for 60 min and stimulated with anti-IgM prior to the read. Tunicamycin was used as a negative control. Each symbol represents the mean value derived from 10 primary, treatment-naïve CLL samples at the indicated time point. Significance was evaluated using a one-way ANOVA followed by Sidak’s multiple comparison test. **C**. Docirbrutinib inhibited BTK and PLCγ2 phosphorylation in treatment-naïve CLL cells. CLL cells were incubated with indicated BTKi for 24 h and protein extracts were made and analyzed using immunoblot for phospho and total proteins. **D**, **E**. Docirbrutinib inhibited spontaneous (**D**) and CXCL12-induced (**E**) migration in primary CLL cells. Cells were treated for 48 h, followed by migration analysis at 5 h. The lower chamber medium contained 100 ng/mL CXCL12. **F**, **G**. BTKi induced modest apoptosis in treatment-naïve (**F**) and relapsed/refractory (**G**) primary CLL cells. Cells were treated for 72 h with BTKi at equimolar concentrations, and annexin V/propidium iodide (PI)–negative events were analyzed. **H**–**J**. Dose-dependent cell death with BTKi in CLL cells with mutant BTK. The primary cells from three patients with CLL with relapse after treatment with cBTKi and ncBTKi were incubated with increasing concentrations of the drugs to generate a dose-response curve. Mean absorbance change was measured in duplicates using resazurin cell viability assay after 72 h of incubation, and results were normalized to DMSO. Docir, docirbrutinib; ns, not significant; Pirto, pirtobrutinib; RFU, relative fluorescence units. Data are expressed as the mean ± the standard deviation. The significance of results was evaluated using a paired, 2-tailed Student *t*-test. *p < 0.05, **p < 0.01, ***p < 0.001, **** *p* < 0.0001.

After validation of docirbrutinib’ s effect in the enzymatic and cell line models, we evaluated the impact on CLL cells from treatment naïve and relapsed/refractory CLL patients. The inhibition of B-cell activation was assessed by measuring surface CD86 marker, a co-stimulatory molecule involved in the activation of B-cells, in CLL cells from 14 treatment-naive patients. Docirbrutinib at 0.01 µM effectively inhibited B-cell activation, yielding results similar to those with ibrutinib and pirtobrutinib at 1 µM (Fig. 3A**)**. Docirbrutinib showed a U-shaped dose-response in this assay, with 0.01–0.1 μM outperforming 1 μM, a pattern we attribute to assay-specific kinetic at higher concentration rather than weaker BTK pathway inhibition. Furthermore, intracellular calcium release was effectively inhibited at docirbrutinib doses of 0.01 µM, 0.1 µM, and 1 µM in primary CLL cells (*n* = 10), with all doses having an inhibitory effect similar to that of ibrutinib and pirtobrutinib at 1 µM (Fig. 3B). The impact of docirbrutinib on the BCR signaling pathway revealed reduced phosphorylation of BTK^Y223^ and downstream PLCγ2^Y1217^ (Fig. 3C). Docirbrutinib at 0.1 µM and 1 µM significantly inhibited the spontaneous migration of primary CLL cells from 10 patients (Fig. 3D). The extent of inhibition observed with docirbrutinib was comparable to that achieved with ibrutinib but much better than that achieved with pirtobrutinib. Furthermore, docirbrutinib suppressed the CXCL12 chemokine–induced migration of primary CLL cells in a dose-dependent manner, with effects similar to those of ibrutinib at 1 µM (Fig. 3E). A mild transient recovery in migration was observed at 4–5 h, followed by a subsequent inhibition by 6 h (Supplemental Fig. S1). Despite prolonged incubation time of 72 h, docirbrutinib at 1 µM resulted in only a moderate increase in apoptosis in CLL cells from 10 treatment-naïve and 16 relapsed/refractory patients (Fig. 3F, G, respectively). The percentage of annexin V/propidium iodide–negative events induced by docirbrutinib at 1 µM was similar to that observed with ibrutinib at 1 µM and pirtobrutinib at 1 µM. The cell death mechanism was identified as caspase dependent, as induction of apoptosis by the combination of higher dose docirbrutinib and venetoclax was completely reversed upon the addition of QVD, a caspase inhibitor (Supplemental Fig. S2). A resazurin cell viability assay was employed in CLL patients who had progression during both cBTKi and ncBTKi treatment and developed BTK mutations such as C481R, L528W, and T474I. Docirbrutinib was effective in various BTK mutants, including in patients who also had mutations in BCL-2, and whereas docirbrutinib was effective against L528W, pirtobrutinib was not (Fig. 3H-J). Furthermore, docirbrutinib inhibited the BCR pathway in WT and C481S + T474I double mutants (Supplemental Figs. S3A and S3B). Docirbrutinib in combination with ibrutinib or pirtobrutinib did not yield any additional effects beyond those observed with each inhibitor alone (Supplemental Fig. S3C and S3D). This finding implies that all agents effectively saturate the binding sites on BTK and do not provide any synergistic advantages when administered concurrently.

### Docirbrutinib administration to patients with CLL inhibits the BCR pathway

**Fig. 4:**
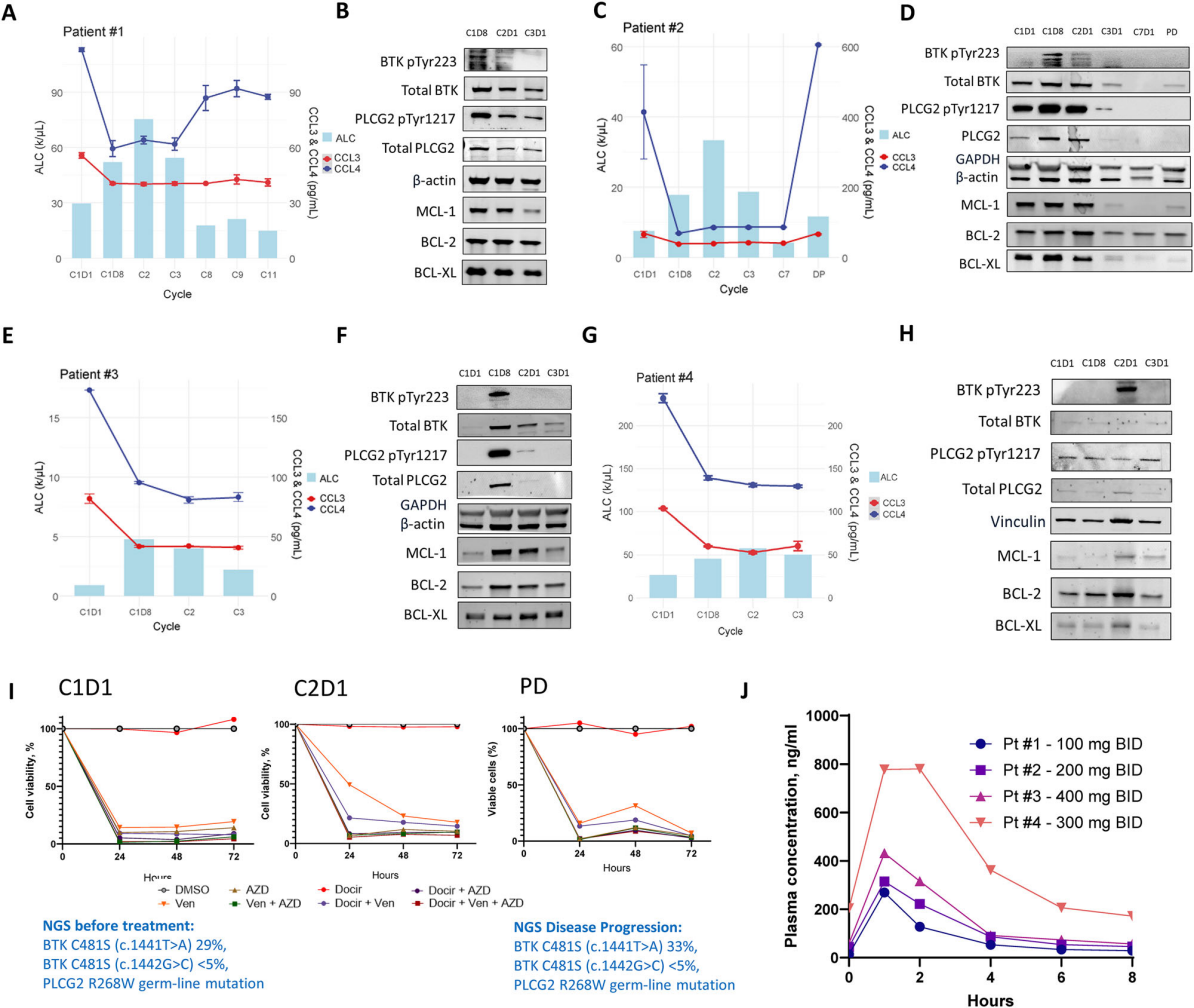
Docirbrutinib inhibited CCL3/CCL4 chemokine levels and the BCR pathway in patients in the phase I clinical trial. **A**, **C**, **E**, **G**. Changes in blood count and chemokine levels during therapy. Peripheral blood samples were collected prior to therapy (C1D1) and at 1 week (C1D8), 4 weeks (C2D1), and 8 weeks (C3D1) after the start of docirbrutinib and at later time points in some patients. Plasma/cell pellets were used for chemokine and immunoblot analysis. CCL3/CCL4 chemokine plasma concentration (measured in duplicate) was superimposed on absolute lymphocyte count retrieved from clinical labratory studies during visit. Data are expressed as the mean ± the standard error of the mean. **B**, **D**, **F**, **H**. Changes in expression of BCR pathway proteins and BCL-2 family proteins during docirbrutinib therapy. Peripheral blood samples were obtained at designated times during therapy and PBMCs were isolated. Protein extracts were made and analyzed using immunoblot for phospho and total proteins of BCR pathway and survival proteins. **I**. Pharmacological profiling from patient #2. Ex vivo drug profiling of patient 2 before treatment, during docirbrutinib therapy, and after disease progression. Measurements are shown across sequential time points for multiple treatment groups, each assessed in a single replicate. **J**. Plasma pharmacokinetics of docirbrutinib during therapy. Blood samples were collected at indicated times on C1D8, and plasma was analyzed for docirbrutinib levels. Pharmacokinetic measurements were obtained from a single plasma sample per subject at each scheduled time point. AZD, AZD5991; BID, twice a day; CxDx, cycle x day x; Docir, docirbrutinib; NGS, next-generation sequencing; Ven, venetoclax.

Biomarker analysis of four patients during the phase Ib clinical trial of docirbrutinib revealed a rapid decrease in plasma levels of the chemokines CCL3 and CCL4 upon treatment initiation, with a decline as early as cycle 1 day 8 (Fig. 4A, C, E, and G). Notably, CCL4 was more responsive than CCL3 to changes in disease status. CCL3 and CCL4 levels negatively correlated with the absolute lymphocyte count, and transient lymphocytosis was observed in all patients. The absolute lymphocyte count also correlated with the level of pBTK and pPLCG2 during treatment. At baseline (C1D1), BCR signaling and BTK expression in peripheral blood CLL cells were modest. Following initiation of docirbrutinib, at C1D8 and C2D1, we observed increased pBTK and pPLCG2 signals (Fig. 4B, D, F, and H), most likely reflecting treatment-induced lymphocytosis and the egression of CLL cells from lymph nodes, where BCR signaling is more active, into the peripheral blood (light blue bars in Fig. 4). With continued docirbrutinib exposure, pBTK and pPLCG2 levels declined, consistent with suppression of BCR signaling at later time points. Additionally, docirbrutinib reduced the expression of total MCL-1, BCL-2, and BCL-XL anti-apoptotic proteins by cycle 3 day 1. Patient 2 experienced disease progression after 7 cycles, accompanied by a substantial increase in plasma CCL3 and CCL4 chemokines, and BTK and MCL-1 expression levels (Fig. 4C and D). However, this patient retained sensitivity to both BCL-2 and MCL-1 inhibitors (Fig. 4I), indicating that these pathways remained viable therapeutic targets. No new BTK mutations were detected in patient 2 at the time of disease progression, leaving the underlying mechanism of resistance in this patient unclear and warranting investigations.

Plasma pharmacology data showed dose-dependent differences in peak plasma level (Supplemental Fig. S4). At a dose of 300 mg, 8 h after oral ingestion of docirbrutinib, the docirbrutinib concentration was approximately 200 ng/mL (Fig. 4J), which is higher than the desired concentration to inhibit the BCR pathway.

### Docirbrutinib exhibits strong synergy with venetoclax and AZD5991 in cell lines and additive effect in primary CLL cells with WT BTK

**Fig. 5:**
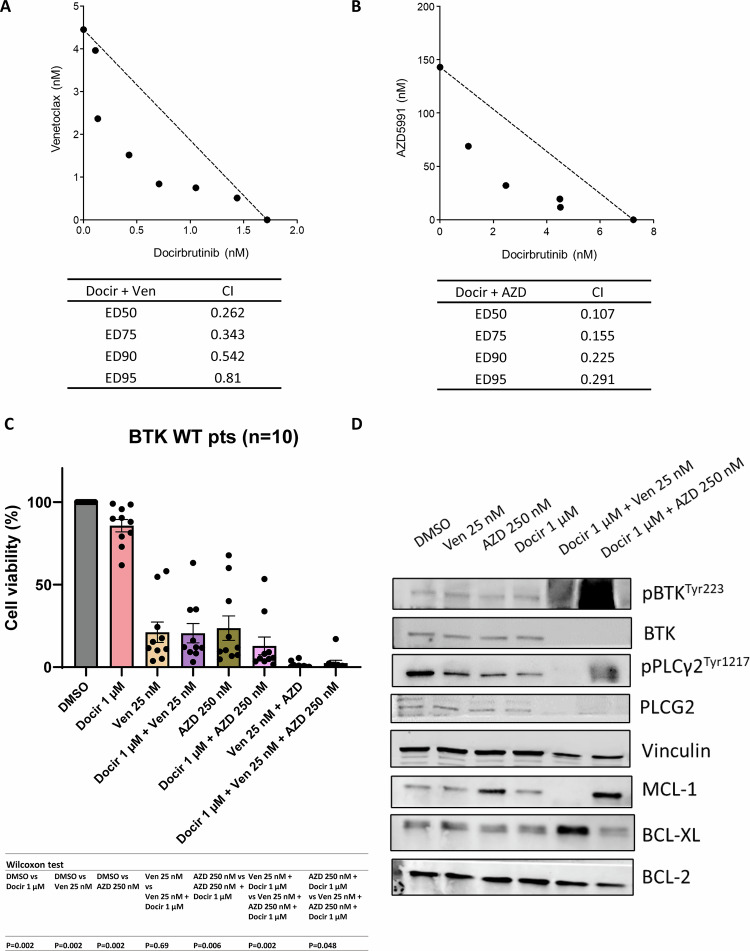
Docirbrutinib was synergistic with BCL-2 inhibitor and MCL-1 inhibitor in cell lines and primary CLL cells with WT BTK. **A**, **B** Testing combinations in OCI-Ly10 cell line. Strong synergism between docirbrutinib and venetoclax (**A**) and between docirbrutinib and AZD5991 (**B**) was observed by isobologram analysis. OCI-Ly10 cells were treated with different concentrations of docirbrutinib and venetoclax or docirbrutinib and AZD5991 for 96 h, and then cell viability was determined by resazurin assay. **C**. Testing combinations in primary CLL cells. Freshly isolated CLL cells from 10 previously treated patients with no BTK mutation were incubated ex vivo with docirbrutinib, venetoclax, and AZD5991 alone and in combination. The apoptosis rate was evaluated by annexin V/propidium iodide staining every 24 h. Statistical comparison between single-agent and combination treatments was performed at the 72 h time point using a paired Wilcoxon test **D**. BTK phosphorylation after ex vivo incubation with docirbrutinib, ibrutinib, and pirtobrutinib. Cells were incubated with single drugs or combinations, proteins were extracted and evaluated by Western blotting. AZD, AZD5991, Docir, docirbrutinib, Ven, venetoclax, CI, Combination index, ED, effective dose.

As the combination of ibrutinib and venetoclax is synergistically effective in preclinical [18–20] and clinical settings [21–24], we examined the effects of combining docirbrutinib and venetoclax and MCL-1 inhibitor AZD5991 in OCI-Ly10 cells and primary CLL cells. To determine whether the combinations are additive or synergistic, we performed isobologram analysis and combination index (CI) calculations based on cell proliferation assay. The isobologram indicated a synergistic effect between docirbrutinib and either venetoclax or AZD5991, as the dose combinations fell below the line of additivity (Fig. 5A and B, Supplemental Fig. S5). The CI values for these combinations were consistently below 1 across all effective doses, further supporting the synergistic effects. In primary CLL cells from ten relapsed patients, drug responses were evaluated using apoptosis assays, as these cells largely quiescent ex vivo and do not proliferate outside their supportive microenvironment and BCR stimulation. Under these conditions, docirbrutinib did not significantly enhance the sensitivity of WT BTK cells to venetoclax (venetoclax alone vs venetoclax plus docirbrutinib at 72 h, *p* = 0.15), since cells were already highly sensitive to venetoclax alone. For AZD5991, the addition of docirbrutinib increased apoptosis compared to AZD5991 alone (72 h, *p* = 0.06). Although this difference did not reach statistical significance, the data indicate a clear trend toward enhanced activity with the combination. BCL-2 and MCL-1 inhibitors combination proved highly lethal to CLL cells, in both the presence (72 h mean viability 2.4%) and absence of BTKi (72 h mean viability 1.3%) (Fig. 5C). Addition of docirbrutinib to venetoclax or AZD5991 significantly inhibited BTK and PLCγ2 phosphorylation (Fig. 5D). Levels of antiapoptotic proteins remained similar except for an increase in MCL-1 protein level with AZD5991 treatment.

### Docirbrutinib in combination with venetoclax is effective against cell lines and primary CLL cells with mutant BTK

**Fig. 6:**
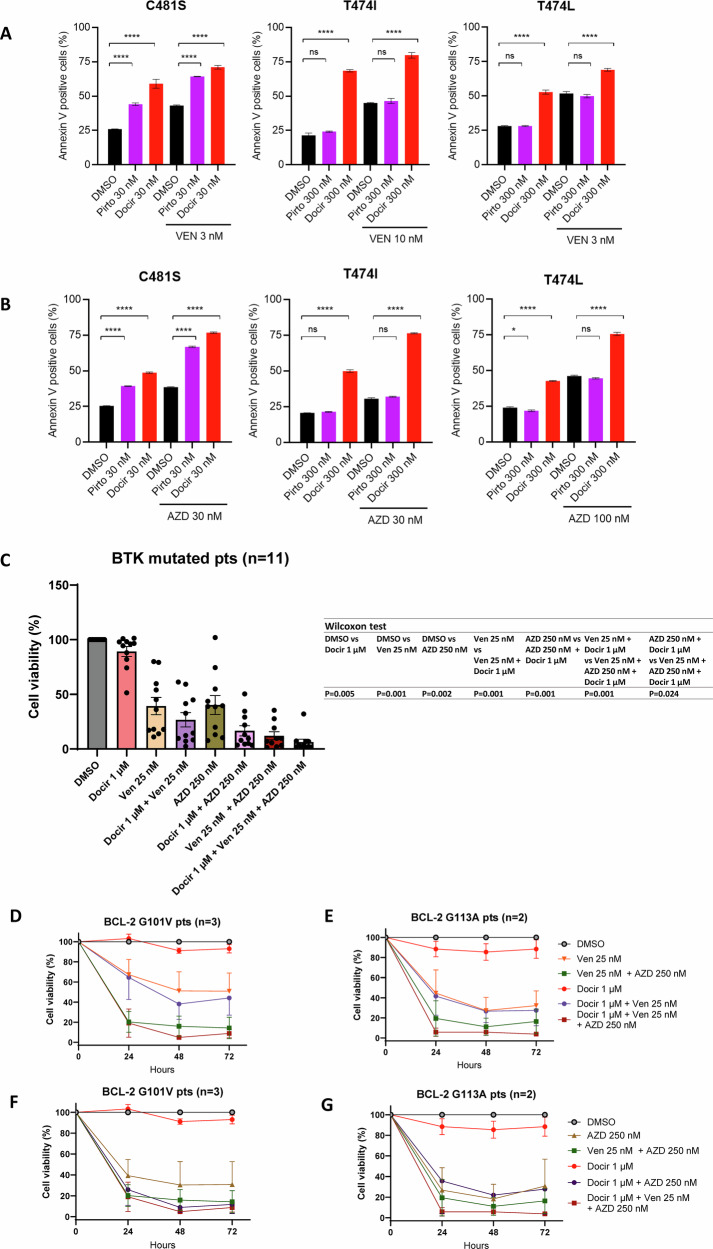
Docirbrutinib was effective when combined with BCL-2 inhibitor or MCL-1 inhibitor in cell lines and primary CLL cells harboring mutant BTK. **A**, **B** Testing combinations in OCI-Ly10 cells with mutant BTK. Effects of the combination of docirbrutinib with venetoclax (**A**) or AZD5991 (**B**) on the induction of apoptosis against OCI-Ly10 cells harboring BTK mutations. OCI-Ly10 cells were treated with compounds for 72 h, stained with 7-AAD/annexin V, and analyzed by flow cytometry. Significance was evaluated using a one-way ANOVA followed by Sidak’s multiple comparison test. ****P < 0.0001, ns: not significant. **C** Testing combinations in primary CLL cells with mutant BTK. CLL cells from eleven patients harboring mutant-BTK were incubated ex vivo with docirbrutinib, venetoclax, and AZD5991 alone and in combination. The apoptosis rate was evaluated by annexin V/propidium iodide staining. Statistical comparison between single-agent and combination treatments was performed at the 72 h time point using a paired Wilcoxon t-test **D**–**G**. Testing combinations in primary CLL cells with mutant BCL-2. Cell viability was measured at 24, 48, and 72 h following treatment in a limited number of samples (BCL-2 G101V, *n* = 3; G113A, *n* = 2). BCL-2 G101 mutation exhibited lower sensitivity to venetoclax (**D**) and AZD5991 (**F**) treatment while G113A-mutated cells remained sensitive to pro-apoptotic drugs (**E**, **G**). Addition of docirbrutinib resensitized cells to venetoclax and AZD5991 in BCL-2 G101V cells. AZD, AZD5991; Docir, docirbrutinib; Pirto, pirtobrutinib; Ven, venetoclax.

We next evaluated apoptosis induced by docirbrutinib combined with venetoclax or AZD5991 in BTK-mutant OCI-Ly10 cells. Superiority of docirbrutinib over pirtobrutinib against mutant BTK appears to be maintained in combinations with BH3 mimetics. Combination treatment with docirbrutinib and either venetoclax or AZD5991 induced significant apoptosis in all BTK-mutant cells, whereas pirtobrutinib combinations were effective only in C481S-mutant cells (Fig. 6A and B). The effect of the combination on apoptosis induction in OCI-Ly10 cells harboring BTK mutations appears to be additive, whereas in OCI-Ly10 cells with wild-type BTK, the combination effect was synergistic, as described above.

While our findings above showed that WT BTK primary CLL samples were sensitive to venetoclax and AZD5991 (Fig. 5C), mutant BTK samples exhibited reduced sensitivity to these agents (Fig. 6C). Docirbrutinib resensitized the mutant BTK cells to pro-apoptotic drugs (Fig. 6C, Supplemental Fig. S6). The response of BCL-2-mutated samples was diverse, and BCL-2 G101V demonstrated lower sensitivity to venetoclax and AZD5991 than BCL-2 G113A did. However, docirbrutinib partially restored the sensitivity to these drugs (Fig. 6D-G).

## Discussion

cBTKi such as ibrutinib, acalabrutinib, and zanubrutinib bind irreversibly to the C481 residue of BTK, resulting in permanent inactivation of BTK. Paradoxically, this desired feature also results in an undesired outcome: mutation of the C481 residue. In contrast, ncBTKi do not rely on the C481 residue for binding and therefore rarely induce C481 mutations. Docirbrutinib is a novel ncBTKi with a slow off-rate, a desirable pharmacological property that enables sustained BTK inhibition similar to cBTKi, leading to prolonged therapeutic effects. Other ncBTKi, such as pirtobrutinib and ARQ531, can inhibit WT and C481-mutant BTK but not mutations at the gatekeeper residue like T474I, which confer resistance to both cBTKi and ncBTKi. Further, kinase-impaired mutations such as L528W have been reported after use of pirtobrutinib [12, 14, 16]. Hence, another desired feature of a new BTKi would be the ability of target both WT BTK and a broader range of mutations. The profiling of docirbrutinib with the panel of BTK mutants revealed that docirbrutinib possesses such broad activity against resistant mutants in vitro and cellular assays. Interestingly, the treatment of kinase-impaired BTK L528W mutant cells with docirbrutinib resulted in significant growth inhibition, whereas ibrutinib and pirtobrutinib showed only limited effects. Proteolytic degradation of BTK L528W mutant by a BTK degrader has been shown to suppress cell survival by disrupting BTK’s scaffold function. Similarly, the tight binding of docirbrutinib to the inactive conformation of BTK L528W may interfere with its scaffold role. Docirbrutinib effectively blocked BCR signaling and reduced the production of chemokines CCL3 and CCL4 in kinase-dead BTK L528W mutant cells, supporting our proposed mechanism involving disruption of scaffold function within the signalosome complex. The docking study using the crystal structure of the inactive conformation of BTK revealed that docirbrutinib does not form key interactions with T474, suggesting that mutations at this position are unlikely to affect its binding affinity. Additionally, the docking simulation using a homology model of the BTK L528W mutant showed no steric hindrance between docirbrutinib and the bulky tryptophan, indicating that docirbrutinib can still be accommodated at the same binding site. Collectively, these findings support that docirbrutinib is effective against both WT and broader range of mutations, including those in the gatekeeper T474 residue of BTK and kinase-dead L528W mutations. In addition to catalytic BTK inhibition, next-generation approaches that target BTK for degradation are emerging as a promising strategy to overcome both kinase-dependent and scaffold-mediated resistance [46–50].

BTK signaling is strongly shaped by lymph node and bone marrow microenvironmental cues, including chronic BCR engagement and accessory survival pathways [51, 52]. Consistent with this concept, longitudinal samples from patients enrolled in the clinical trial showed that following treatment-induced lymphocytosis, circulating CLL cells displayed increased BTK signaling and elevated expression of BCL-2 family pro-survival proteins. This pattern likely reflects the mobilization of CLL cells from protective lymphoid niches, where microenvironmental signals sustain active BCR signaling and survival programs. Previous studies have shown that BTK inhibition broadly disrupts microenvironment-dependent processes in CLL. In murine models, BTK inhibition reduces CXCR4 surface expression and downstream signaling, leading to impaired homing and retention of CLL cells and their redistribution into the peripheral blood. [53] Similar effects were observed in patients during therapy [54] In addition, in vivo analyses demonstrated that ibrutinib disrupts tumor-microenvironment interactions by decreasing key chemokines and chemoattractants and reducing CLL cell chemoattraction [55] Given the high selectivity of docirbrutinib, we anticipate that it exerts comparable effects on chemokine receptor expression, cellular trafficking, and microenvironmental signaling, further contributing to CLL cell mobilization and therapeutic vulnerability. As additional patient samples are being collected, we plan to assess dynamic changes in CXCR4 expression during therapy to further define the impact of docirbrutinib on microenvironmental interactions.

Although BTKi monotherapy prolongs progression-free survival, it rarely achieves complete remission [4, 11]. Hence, it became critical to evaluate combinations of BTKi with other agents. Given that BCL-2 and MCL-1 are key survival proteins in CLL cells [28], multiple preclinical studies [18, 19, 31, 56] and clinical trials [21, 24, 35, 57] have evaluated combinations of BTKi with venetoclax, and nanomolecules incorporating both agents have been designed. Considering these successes and the rationale for combining BTKi with BCL-2 or MCL-1 inhibitors, we tested docirbrutinib in combination with venetoclax and AZD5991. Venetoclax is approved for clinical use; AZD5991 is used as a tool compound for preclinical studies. Current clinical MCL-1 inhibitors result in cardiotoxicity but different efforts to create better inhibitors are ongoing. These novel strategies include binding to different pocket of the protein [58], novel scaffolds [59, 60], drug with fast-clearance [61], and degrader [62]. Co-treatment with docirbrutinib and either venetoclax or AZD5991 led to cell viability and the signaling pathway inhibitions. While cell line models demonstrated strong synergy between docirbrutinib and venetoclax or AZD5991, primary CLL cells were already highly sensitive to venetoclax or AZD5991 alone, with docirbrutinib providing primarily additive effects; the influence of distinct microenvironmental contexts on these responses remains an important area for future investigation. These data underscore the need for a clinical trial of the combination of docirbrutinib and venetoclax for CLL.

Docirbrutinib is being evaluated in a first-in-human phase I clinical trial for B-cell malignancies, including CLL [37]. Fourteen patients have been enrolled, including nine with CLL. The starting dose was 100 mg orally BID, and the dose is being escalated to reach a desired dose with biological activities [37]. Doses of greater than 300 mg BID resulted in the desired plasma concentration ( > 50 ng/mL) to block BCR signaling. However, even at doses of 100 mg BID and 200 mg BID, we observed inhibition of BCR pathway signaling; decrease in CCL3, a biomarker of response; and decline in tumor cells.

Since 2013 [63], BTKi use has become ubiquitous in CLL treatment, for both treatment-naïve and relapsed patients [4]. As a result, there is increasing number of patients with CLL who have resistance to BTKi with mutations in BTK and need additional therapy. Having a BTKi such as docirbrutinib that combines qualities of cBTKi and ncBTKi can address this limitation. Similarly, there is a need for a BTKi that targets various mutant BTK irrespective of the mutational site, i.e., kinase domain, gatekeeper, and non-kinase domain. This demand has driven the development of novel BTKi with dual cBTKi and ncBTKi, such as LP168 [64]. We found in cell-free assays and *in vitr*o cellular assays that docirbrutinib inhibited both WT BTK and various mutant BTK. Such a pan-BTKi is critically needed for patients who have CLL resistant to cBTKi and other ncBTKi. The ongoing clinical trial will establish the efficacy of docirbrutinib for patients as well as its impact on CLL cells with mutant BTK. The protocol has been amended to include patients who were previously treated with pirtobrutinib and have disease progression. Finally, the results of our preclinical evaluation justify use of docirbrutinib in combination with venetoclax for previously treated patients harboring BTK mutations.

## Supplementary information

Supplemental Methods

Supplemental Tables

Supplemetal Figures

AJ checklist

## Data Availability

Enzymatic and cell line experimental data generated in this study are available from the Carna Biosciences, Inc. upon reasonable request. Raw experimental data from treatment-naïve and relapsed CLL patients outside the clinical trial may be made available from corresponding author upon request following anonymization, with the exception of genetic data. Access to the individual participant data enrolled in the NCT05602363 clinical trial will follow the statement in the future publication of clinical trial results.
