# Supplementary material for: Docirbrutinib is a pan-mutant BTK inhibitor and inhibits B-cell receptor signaling in chronic lymphocytic leukemia cells in preclinical and early clinical investigations
Source: Blood Cancer J. 2026 May 7;16(1):107. doi: 10.1038/s41408-026-01509-8 (PMC13319122; doi:10.1038/s41408-026-01509-8)
Supplement: Supplementary file 1 — Supplemental Methods [file 41408_2026_1509_MOESM1_ESM.docx]

**Supplemental Methods**

**ADP-Glo Max Assay**

Recombinant biotinylated human BTK enzymes with N-terminal DYKDDDDK tags were obtained from the Drug Discovery Support Business Division of Carna Biosciences, Inc. (Details in **Supplemental Table 5**). Srctide peptide substrate (RGEEPLYWSFPAKKK-NH2) was synthesized by GenScript (Tokyo, Japan). 4× compound solution, 4× substrate mixture solution containing Srctide, ATP and MgCl2 , and 2× kinase solutions were prepared by dilution with ADP-Glo Max assay buffer. For kinase assay, a mixture of compound solution and kinase solution was pre-incubated and then substrate mixture solution was added to the mixture and incubated for the appropriate time at room temperature (Details in **Supplemental Table 6**). The kinase reaction was terminated by adding ADP-Glo Reagent, and ADP production was measured using ADP-Glo Max Detection Reagent. Data were acquired with an EnVision XCite multilabel plate reader (Revvity). All assays were performed in duplicate, and the inhibition rate (%) of the test compound was calculated according to the following formula:

$$％ inhibition=\left\{ 1-\frac{RL{U\_mean}_{\left[ experimental \right]}-RL{U\_mean}_{\left[ Negative \right]}}{RL{U\_mean}_{\left[ Positive \right]}-RL{U\_mean}_{\left[ Negative \right]}} \right\}\times100$$

*RLU* indicates relative light units. RLU_mean_[experimental]_: mean RLU of compound-added sample. RLU_mean_[Positive]_: mean RLU of DMSO-added sample. RLU_mean_[Negative]_: mean RLU of no-kinase sample. The IC_50_ values were calculated by nonlinear regression analysis of the inhibition rate and the test compound concentration (logarithm) using GraphPad Prism 5.

**LanthaScreen Eu kinase binding assay**

The competitive binding assay was performed according to the manufacturer’s protocol. Briefly, BTKi was added to a mixture of Kinase Tracer 178, Eu-labeled streptavidin, and BTK enzyme solution and incubated for 1 hour at room temperature (Details in **Supplementary Table 7**). Data were acquired with an EnVision XCite multilabel plate reader at excitation and emission wavelengths of 340 nm and 615 nm and 665 nm, respectively. The inhibitory potency of the test compound was calculated using the following formula:

$$Emission ratio=\frac{emission signal at 665 nm}{emission signal at 615 nm}$$

$$％inhibition=\left\{ 1-\frac{[Emission ratio\_mean]-[Tracer(-) Emission ratio\_mean]}{[Tracer(+) Emission ratio\_mean]-[Tracer(-) Emission ratio\_mean]} \right\}\times100$$

Emission ratio_mean: mean emission ratio of compound-added sample. Tracer(+) Emission ratio_mean: mean emission ratio of DMSO-added sample. Tracer(-) Emission ratio_mean: emission ratio of no-tracer sample.

All assays were performed in duplicate, and the IC_50_ values were calculated by nonlinear regression analysis of the inhibition rate and the test compound concentration (logarithm) using GraphPad Prism 5.

**Cell culture**

HEK293 cells were purchased from ATCC (Manassas, VA, USA). They were cultured in DMEM high-glucose medium (Nacalai, Kyoto, Japan) supplemented with 10% heat-inactivated FBS (Sigma-Aldrich, St. Louis, MO, USA) and 1% penicillin-streptomycin (Nacalai). OCI-Ly10 cells were obtained from University Health Network, Ontario, Canada. These cells were cultured in IMDM medium (Invitrogen, Waltham, MA, USA) supplemented with 20% heat–non-inactivated FBS (Hyclone, Logan, UT, USA) and 1% penicillin-streptomycin. Cells were routinely tested for mycoplasma contamination and authenticated by short tandem repeat (STR) profiling prior to use.

**pBTK inhibition assay in HEK293 cells**

HEK293 cells were transfected with the appropriate BTK expression plasmid (pcDNA4_V5-His-BTK WT, pcDNA4_V5-His-BTK C481S, or pcDNA4_V5-His-BTK T474I) using TransIT-LT1 transfection reagents (Takara-Bio, Shiga, Japan), and the corresponding stable cell lines were established in the presence of Zeocin (Thermo Fisher Scientific, Waltham, MA, USA). For other BTK mutants (T474L, T474M, T474I/C481S, T474M/C481S, T474M/C481T, L528M, and L528V), HEK293 cells were transduced with a retroviral vector encoding the corresponding BTK mutant, and cells with mutations were selected in the presence of Puromycin (Invivogen, San Diego, CA, USA).

HEK293 cells expressing the appropriate BTK mutant were treated with BTK inhibitors at various concentrations at 37℃ for 24 hours. Then, the cells were washed with D-PBS (Nacalai) and lysed using a lysis buffer (Cell Signaling Technology, Danvers, MA, USA) containing 1% phosphatase inhibitor cocktail (Nacalai), 1% phosphatase inhibitor cocktail 3 (Sigma-Aldrich), and 1 mM phenylmethylsulfonyl fluoride (Nacalai). The supernatant was collected by a centrifugal operation at 14,500 rpm for 15 minutes. The obtained samples were analyzed by Western blotting. The primary antibodies used in this experiment were as follows: anti-total BTK (BD Transduction Laboratories, New Jersey, USA, RRID:AB_398427), anti-phospho Thy223 BTK (Abcam, Cambridge, MA, UK, RRID:AB_11155512), and anti-beta actin (Proteintech, Rosemont, IL, USA, RRID:AB_2687938). Band detection was performed using Chemi-Lumi One Super (Nacalai) according to the manufacturer’s protocol. Each band was detected by chemiluminescence using a FUSION-FX7.EDGE imaging system (Vilber Bio Imaging, Marne-la-Vallee, France).

**Next-generation sequencing of clinical samples**

Libraries were generated from genomic DNA through hybridization capture-based enrichment targeting specific genomic regions of interest. Bidirectional paired-end sequencing was conducted on a next-generation sequencing platform to identify single-nucleotide variants and small insertions or deletions. The GRCh37/hg19 genome served as the reference. Genes, exons, and codons with ≥250× coverage were analyzed. Samples required ≥10% tumor content, and the assay's detection limit was 5% mutant allele frequency (one mutant allele in the background of 19 WT alleles). We performed next-generation sequencing of 162 genes associated with B-cell neoplasms (**Supplemental Table S4).**

**Drugs and treatment**

In vitro incubations were performed with docirbrutinib (0.01-10 µM; Carna Biosciences, Inc.), ibrutinib (1 µM; MedChemExpress), acalabrutinib (1 µM; MedChemExpress), pirtobrutinib (1 µM; MedChemExpress), venetoclax (1-50 nM; MedChemExpress), and AZD5991 (10-500 nM; MedChemExpress). For the calcium release assay, tunicamycin 10 mM (Abcam) served as the negative control. For the MitoSOX ROS production assay, LCS-1 (selective superoxide dismutase 1 inhibitor; 1 µM, Selleckchem) and FCCP (oxidative phosphorylation uncoupler; 5 µM, Selleckchem) served as positive controls, while glutathione (antioxidant; 100 mM) served as a negative control. For all drugs, stock solutions were made in DMSO (Sigma). DMSO in matching concentration was used as the vehicle control.

**Cell migration, proliferation, apoptosis, calcium release, MitoSOX and B-cell activation assays**

For measurement of spontaneous and CXCL12-induced migration cells was placed in a transwell insert (Corning) resting in medium either supplemented or not with 100 ng/mL CXCL12 (R&D Systems). After 6 hours incubation the inserts were removed, and the cells migrated through the insert were counted via flow cytometry for 1 minute. Migrated cells count in the lower compartment was normalized to total count of cells seeded in the well. For proliferation cells were treated with the indicated compounds and were stained with resazurin solution and absorbance was measured at 570 nm excitation and 600 nm emission using a BioTek Synergy microplate reader. For apoptosis, CD5/CD19 co-staining was used to identify CLL cells, and FACS analysis was performed on annexin V/propidium iodide-stained cells.

For calcium release assay, primary CLL cells stained with Rhod-2 AM dye (Abcam) and then incubated with DMSO, drugs or tunicamycin (Tocris; negative control). Cells were stimulated by 10 µg/mL anti-IgM (Jackson Immuno; RRID:AB_2337553) and the absorbance was read immediately at 550 excitation and 581 emission every 10 seconds for 15 minutes on a BioTek Synergy plate reader. For B-cell activation, CLL cells were incubated with DMSO or BTKi and stimulated with 10 µg/mL anti-IgM for 24 hours. The cells were then stained with CD86 (RRID: AB_396019) and analyzed via flow cytometry.

**Immunoblot**

For immunoblotting, 30–60 µg of protein from cell lysates was separated on 4–12% gradient criterion protein gels (Bio-Rad) with 3-(N-morpholino) propane-sulfonic acid running buffer (Bio-Rad). Proteins were then transferred onto polyvinylidene difluoride membranes (Bio-Rad). Membranes were segmented and incubated with specific primary antibodies listed in **Supplemental Table 3**, followed by species-specific secondary antibodies conjugated with horseradish peroxidase (Bio-Rad). Protein bands were detected using the Odyssey Infrared Imaging System (LI-COR Biosciences) and ChemiDoc Imaging System (Bio-Rad). Signal intensities were quantified using LI-COR Image Studio Software (RRID:SCR_015795) and normalized to internal loading controls such as β-actin, vinculin, or smooth muscle actin.
