# Supplementary material for: Docirbrutinib is a pan-mutant BTK inhibitor and inhibits B-cell receptor signaling in chronic lymphocytic leukemia cells in preclinical and early clinical investigations
Source: Blood Cancer J. 2026 May 7;16(1):107. doi: 10.1038/s41408-026-01509-8 (PMC13319122; doi:10.1038/s41408-026-01509-8)
Supplement: Supplementary file 2 — Supplemental Tables [file 41408_2026_1509_MOESM2_ESM.docx]

**Supplemental Tables**

**Supplemental Table S1. Patient characteristics of treatment-naïve patients**

| ID | IGHV | FISH | WBC count,  x10^9^/mL | Assays |
| --- | --- | --- | --- | --- |
| 847 | Unmutated | 11Q | 21.7 | A/PI, BCA |
| 099 | Unmutated | NEG | 89.4 | A/PI, BCA, ROS |
| 413 | Mutated | 13Q | 40.9 | A/PI |
| 494 | Unmutated | 11Q & 13Q | 189.6 | A/PI, BCA, Ca2+R , ROS |
| 527 | Unk | NEG | 54 | A/PI |
| 644 | Unmutated | 13Q | 95.4 | A/PI, BCA, ROS |
| 680 | Mutated | NEG | 60.9 | A/PI, BCA, Ca2+R , ROS |
| 836 | Mutated | NEG | 22.5 | A/PI |
| 838 | Unmutated | 13Q | 26.4 | A/PI, BCA, ROS |
| 853 | Mutated | 13Q | 55.6 | A/PI, BCA, ROS |
| 883 | Mutated | 13Q | 40.8 | A/PI, BCA, ROS |
| 308 | Unmutated | 11Q & 13Q | 46.7 | BCA, Ca2+R , ROS |
| 283 | Mutated | 13Q | 82.4 | BCA, Ca2+R , ROS |
| 445 | Mutated | NEG | 44.7 | BCA, Ca2+R |
| 357 | Mutated | 13Q | 119.7 | BCA, Ca2+R , BCM |
| 723 | Mutated | 13Q | 28.4 | BCA |
| 007 | Mutated | 13Q | 62.1 | BCA, Ca2+R , ROS |
| 683 | Unmutated | 17P & TRI12 | 164.3 | Ca2+R |
| 370 | Mutated | 13Q | 33.9 | Ca2+R |
| 758 | Unmutated | 13Q | 28 | Ca2+R |
| 128 | Mutated | 13Q | 69.6 | ROS, BCM |
| 123 | Mutated | 13Q | 72 | ROS |
| 377 | Mutated | 13Q | 57 | ROS, BCM |
| 198 | Mutated | 13Q | 25 | ROS, BCM |
| 864 | Mutated | 13Q | 20.5 | ROS, BCM, CCL3/CCL4 |
| 073 | Mutated | 13Q | 42.4 | BCM |
| 288 | Unmutated | TRI12 | 128.2 | BCM |
| 019 | Unk | NEG | 44.4 | BCM |
| 422 | Unmutated | NEG | 55 | BCM |
| 185 | Unmutated | 13Q | 22.8 | BCM |

A/PI, annexin V/propidium iodide; BCA, B-cell activation; ROS, reactive oxygen species assay; BCM, B-cell migration; Unk, unknown; WBC, white blood cell.

**Supplemental Table S2. Characteristics of previously treated (relapsed/refractory disease) patients.**

| ID | Previous targeted Rx | BTK mutation(s) | Other mutations |
| --- | --- | --- | --- |
| 060 | Pirtobrutinib | BTK C481R, L528W, T474I | R107_R110dup |
| 504 | Pirtobrutinib | L528W |  |
| 964 | Pirtobrutinib | T474I |  |
| 808 | Pirtobrutinib | BTK T474I, L528W | BCL2 G101V, BCL2 R107_R110R, DNMT3A, RPS15, SF3B1, TET2, TP53 |
| 116 | Pirtobrutinib | BTK L528W, T474I | EGR2, NOTCH-1, CHD2, TP53, SPEN, POT1, NF1, PRDM1, MFHAS1 |
| 728 | Ibrutinib + venetoclax,  pirtobrutinib | BTK T474F | BRCC3, EGR2, FAT1, IGLL5, KMT2D, SMARCA4, TET2, TP53, XPO1 |
| 582 | Ibrutinib + venetoclax |  | BCL2 A113G, MYD88 S206C |
| 203 | Ibrutinib + venetoclax, rituximab, CAR-T | BTK C481S | COR, BRAF, DNMT3A, KMT2D, SF3B1, ATM, FAT1, PLCG2 R268W, S1PR1, SP140, ZFAT |
| 660 | Pirtobrutinib + obinutuzumab |  | KMT2D NOTCH1, TP53 G105D, DDX3X, TRAF2A, ASXL1, CIITA, DUSP2, FAT1 , PLCG2 H244R, SPEN |
| 044 | Acalabrutinib + venetoclax, CAR-T | BTK C481R | BCL2 D103E, BCL2 G101V, PLCG2 M114R |
| 983 | Ibrutinib + venetoclax + rituximab |  | BCL2 G110V, BCL2 A76T, DNMT3A, FBXW7, IGLL5, ITPKB, KRAS |
| 038 | Ibrutinib,  pirtobrutinib | BTK C481R | ATM, BCL2 A113G, DIS3, IGLL5, PLCG2, SF3B, TP53, CNOT3, FGER3, POLE, PRDM1 |
| 785 | Ibrutinib + venetoclax + rituximab | BTK C481R,  BTK I432R | ATM, RCC3, ITPKB, TET2 |
| 607 | Ibrutinib + venetoclax + obinutuzumab |  | POT1, SF3B1, ARID1A |
| 528 | Ibrutinib + venetoclax |  | KRAS, SF3B, DDX3X, DUSP2, PAX5, BIRC3 |
| 565 | Ibrutinib |  | TP53, SF3B |
| 185R | Ibrutinib + venetoclax |  | SF3B |
| 958 | Pirtobrutinib + venetoclax + obinutuzumab | BTK T474I, C481S, C481S, T474P | MAP3K14, NOTCH1, SF3B1, MAP3K14, POLE, RB1, TBL1XR1, TP53 c.776_781del, ATM, BCL6, FAT1, KMT2D, POLE, RBMX |
| 288R | Ibrutinib + venetoclax |  | BRAF, SF3B, ELF4, SAMHD1, KMT2D, PLCG2 H257L, PLOE, TET2, TNFRS14 |
| 085 | Ibrutinib + venetoclax |  | BIRC3, RFTN1, SETD2, FA1, IKZF3, KMT2D, NOTCH2, PLCG2, S1PR2, SAMHD1, SPEN |
| 781 | Ibrutinib, acalabrutinib | BTK T474I, C481S | XPO |
| 878 | Ibrutinib + venetoclax,  acalabrutinib,  venetoclax, rituximab  obinutuzumab |  | SF3B1, TP53 R248W, BAZ2A, HUWE1 (Mule1), BAZ2A, BCL6, FAT1, PLCG2 R268W, SMARCA4 |
| 443 | Ibrutinib + venetoclax | BTK C481S | TP53, PTPN11, NF1, MAPK2K1, KRAS, CARD11 |

CAR-T, chimeric antigen receptor T-cell; Rx, therapy.

**Supplemental Table S3. Western blot primary antibodies**

**All proteins except antioxidants**

| **Protein** | **kDa** | **Antibody name** | **Dilution** | **Catalog No., RRID** |
| --- | --- | --- | --- | --- |
| Vinculin | 124 | Vinculin (E1E9V) XP rabbit | 1:1000 | Cell Signaling Technology Cat# 13901, RRID:AB_2728768 |
| PARP | 113 | PARP (46D11) rabbit mAb | 1:1000 | Cell Signaling Technology Cat# 9532, RRID:AB_659884 |
| β-actin | 42 | Anti-beta actin antibody [GT5512] mouse | 1:2000 | GeneTex Cat# GTX629630, RRID:AB_2728646 |
| MCL1 | 40 | Mcl-1 (D2W9E) rabbit mAb | 1:1000 | Cell Signaling Technology Cat# 94296, RRID:AB_2722740 |
| BCL-XL | 30 | Bcl-xL (54H6) rabbit mAb | 1:1000 | Cell Signaling Technology Cat# 2764, RRID:AB_2228008 |
| BCL2 | 26 | Bcl-2 (124) mouse mAb | 1:1000 | Cell Signaling Technology Cat# 15071, RRID:AB_2744528 |
| BTK | 76 | Btk (D6T2C) Mouse mAb | 1:1000 | Cell Signaling Technology Cat# 56044, RRID:AB_2799503 |
| p-BTK | 76 | Anti-phospho-BTK (pTyr^223^) antibody produced in rabbit | 1:1000 | SAB4503801-100UG, RRID: AB_3676293 |
| PLCγ2 | 155 | PLCG2 monoclonal antibody mouse | 1:1000 | Proteintech Cat# 67011-1-Ig, RRID:AB_2882328 |
| p-PLCγ2 | 155 | Phospho-PLCγ2 (Tyr1217) antibody rabbit | 1:1000 | Cell Signaling Technology Cat# 3871, RRID:AB_2299548 |
| GADPH | 36 | GAPDH (D4C6R) mouse mAb | 1:1000 | Cell Signaling Technology Cat# 97166, RRID:AB_2756824 |

mAb, monoclonal antibody.

**Supplemental Table S4. EndLymphoma panel gene list**

ARID1A, ASXL1, ATM, B2M, BAZ2A, BCL10, BCL2, BCL6, BCL7A, BCOR, BIRC3, BLNK, BRAF, BRCC3, BTG1, BTG2, BTK, CARD11, CCND1, CCND3, CCR4, CCR7, CD274, CD28, CD58, CD79A, CD79B, CDKN2A, CDKN2B, CHD2, CHEK2, CIITA, CNOT3, CREBBP, CXCR4, DDX3X, DIS3, DNMT3A, DUSP2, EGR1, EGR2, ELF4, EP300, EWSR1, EZH2, FAM50A, FAS, FAT1, FBXW7, FGFR3, FOXO1, FYN, GNA13, GNAS, GPR183, H1-2, H1-4, H3C2, HRAS, HUWE1, HVCN1, ID3, IDH1, IDH2, IFNGR1, IGLL5, IKZF3, IL2RG, IRAK1, IRF4, IRF8, ITPKB, JAK1, JAK2, JAK3, KIT, KLF2, KLHL6, KMT2D, KRAS, LTB, LYN, MAP2K1, MAP3K14, MAPK1, MAX, MED12, MEF2B, MFHAS1, MYC, MYD88, NF1, NFKB2, NFKBIA, NFKBIE, NOTCH1, NOTCH2, NPM1, NRAS, NSD2, NXF1, P2RY8, PAX5, PCBP1, PIK3CA, PIK3R1, PIM1, PLCG1, PLCG2, PLEKHG5, POLE, POT1, PRDM1, PTEN, PTPN1, PTPN11, PTPRD, RASSF1, RB1, RBMX, RFTN1, RHOA, RIPK1, RPS15, RRAGC, RRAS, S1PR1, S1PR2, SAMHD1, SETD2, SF3B1, SGK1, SMARCA4, SMO, SOCS1, SOX11, SP140, SPEN, SRSF2, STAT3, STAT5B, STAT6, STK11, SYK, TBL1XR1, TCF3, TENT5C, TET2, TMEM30A, TNFAIP3, TNFRSF14, TP53, TRAF2, TRAF3, TRAF6, U2AF1, UBR5, VAV1, XPO1, ZFAT, ZMYM3, ZRSR2

**Supplemental Table S5. List of recombinant BTK mutant proteins**

| Mutation | Enzymatic activity | Assay format | Carna Biosciences  catalog no. | Ref# |
| --- | --- | --- | --- | --- |
| T316A | yes | ADP-Glo | 08-418-23N | 1 |
| C481S | yes | ADP-Glo | 08-417-23N | 2 |
| T474I | yes | ADP-Glo | 08-419-23N | 3 |
| T474S | yes | ADP-Glo | 08-420-23N | 4 |
| T474M | yes | ADP-Glo | 08-916-23N | 4 |
| T474L | yes | ADP-Glo | 08-920-23N | 5 |
| T474M/C481S | yes | ADP-Glo | 08-917-23N | 4 |
| T474I/C481S | yes | ADP-Glo | 08-918-23N | 4 |
| T474M/C481T | yes | ADP-Glo | 08-919-23N | 4 |
| L528W | no | TR-FRET | 08-915-20N | 3 |
| L528M | yes | ADP-Glo | 08-912-23N | 6 |
| L528V | yes | ADP-Glo | 08-914-23N | 6 |
| L528S | no | TR-FRET | 08-913-20N | 7 |
| L528F | no | TR-FRET | 08-911-20N | 6 |

TR-FRET indicates time-resolved fluorescence resonance energy transfer. The assay was performed using the commercial LanthaScreen Eu kinase binding assay. T474M, T474L, T474M/C481S, T474I/C481S, T474M/C481T, L528W, L528M, L528V, L528S and L528F mutants were obtained by custom production services at Carna Biosciences.

References:

1. Sharma S, Galanina N, Guo A, et al. Identification of a structurally novel BTK mutation that drives ibrutinib resistance in CLL. Oncotarget. 2016;7(42):68833-68841.
2. Woyach JA, Ruppert AS, Guinn D, et al. BTKC481S-Mediated Resistance to Ibrutinib in Chronic Lymphocytic Leukemia. J Clin Oncol. 2017;35(13):1437-1443.
3. Wang E, Mi X, Thompson MC, et al. Mechanisms of Resistance to Noncovalent Bruton's Tyrosine Kinase Inhibitors. N Engl J Med. 2022;386(8):735-743.
4. Estupiñán HY, Wang Q, Berglöf A, et al. BTK gatekeeper residue variation combined with cysteine 481 substitution causes super-resistance to irreversible inhibitors acalabrutinib, ibrutinib and zanubrutinib. Leukemia. 2021;35(5):1317-1329.
5. Naeem A, Utro F, Wang Q, et al. Pirtobrutinib targets BTK C481S in ibrutinib-resistant CLL but second-site BTK mutations lead to resistance. Blood Adv. 2023;7(9):1929-1943.
6. Possible mutants predicted by single-nucleotide change in the codon for L528.
7. Qi J, Endres S, Yosifov DY, et al. Acquired BTK mutations associated with resistance to noncovalent BTK inhibitors. Blood Adv. 2023;7(19):5698-5702.

**Supplemental Table S6. Assay conditions for ADP-Glo Max assay**

| BTK protein | | Srctide substrate, | ATP, | MgCl_2_, | Reaction time, min |
| --- | --- | --- | --- | --- | --- |
| Mutation | Conc., nM | Conc., μM | Conc., mM | Conc., mM |  |
| WT | 1.2 | 150 | 1 | 5 | 60 |
| T316A | 1.0 | 150 | 1 | 5 | 60 |
| C481S | 1.2 | 150 | 1 | 5 | 60 |
| T474I | 1.2 | 150 | 1 | 5 | 60 |
| T474S | 2.0 | 150 | 1 | 5 | 60 |
| T474M | 1.3 | 150 | 1 | 5 | 80 |
| T474L | 1.3 | 150 | 1 | 5 | 80 |
| T474M/C481S | 1.3 | 150 | 1 | 5 | 80 |
| T474I/C481S | 1.3 | 150 | 1 | 5 | 80 |
| T474M/C481T | 1.3 | 150 | 1 | 5 | 80 |
| L528M | 2.4 | 150 | 1 | 5 | 140 |
| L528V | 2.0 | 150 | 1 | 5 | 80 |

Conc., concentration.

**Supplemental Table S7. Assay conditions for LanthaScreen Eu kinase binding assay**

| BTK protein | | Kinase tracer 178, | Eu-streptavidin |
| --- | --- | --- | --- |
| Mutation | Conc., nM | Conc., nM | Conc., nM |
| WT | 2.0 | 4 | 1 |
| L528W | 2.0 | 4 | 1 |
| L528S | 4.0 | 25 | 1 |
| L528F | 2.0 | 4 | 1 |

Conc., concentration.

**Supplemental Table S8. IC_50_ values for autophosphorylation at Tyr223 of BTK in HEK293 cells expressing BTK mutants**

| BTK mutant | pY223 IC_50_, nM | | |
| --- | --- | --- | --- |
|  | Docirbrutinib | Pirtobrutinib | Ibrutinib |
| WT | 4.4 ± 1.8 | 5.1 ± 3.3 | 2.3 ± 0.7 |
| C481S | 1.9 ± 0.5 | 9.3 ± 5.3 | 253.7 ± 82.1 |
| T474I | 6.7 ± 1.9 | 615.8 ± 218.6 | 0.7 ± 0.2 |
| T474M | 5.3 | > 10000 | NT |
| T474L | 13.5 | 5511 | NT |
| L528M | < 0.3 | 9.2 | NT |
| L528V | 1.7 | 39.1 | NT |
| T474I/C481S | 49.4 | 775 | > 10000 |
| T474M/C481S | 20.1 | 3554 | > 10000 |
| T474M/C481T | 11 | 337 | > 10000 |

NT, not tested. The values were expressed as the mean ± SEM of at least three independent experiments.

**Supplemental Table S9. Antiproliferative activity of compounds in OCI-Ly10 cells harboring C481S, T474I, T474L, T474M, L528V or L528W in BTK**

| Mutation^a^ | IC_50_, nM | | |
| --- | --- | --- | --- |
|  | Docirbrutinib | Pirtobrutinib | Ibrutinib |
| WT | 2.5 ± 0.1 | 6.4 ± 1.6 | 1.0 ± 0.1 |
| C481S | 11.5 ± 7.8 | 16.2 ± 3.4 | 813 ± 199 |
| T474I | 65 ± 26 | 1830 ± 439 | 1.6 ± 0.6 |
| T474L | 103.6 ± 4.3 | 4045 ± 439 | 58.6 ± 5.6 |
| T474M | 66.3 ± 8.8 | 4757 ± 1128 | 89.3 ± 18.4 |
| L528V | 5.8 ± 1.3 | 127 ± 33 | 0.3 ± 0.1 |
| L528W | 4.5 ± 1.2 | > 1306 | 25.6 ± 13.7 |

^a^C481S, T474I, T474L, T474M, and L528V mutants: knock-in; L528W mutant: overexpression. The values were expressed as the mean ± SD of at least three independent experiments.
