# Supplementary material for: Docirbrutinib is a pan-mutant BTK inhibitor and inhibits B-cell receptor signaling in chronic lymphocytic leukemia cells in preclinical and early clinical investigations
Source: Blood Cancer J. 2026 May 7;16(1):107. doi: 10.1038/s41408-026-01509-8 (PMC13319122; doi:10.1038/s41408-026-01509-8)
Supplement: Supplementary file 3 — Supplemetal Figures [file 41408_2026_1509_MOESM3_ESM.pdf]

Supplemental Figures

Supplemental Figure 1.

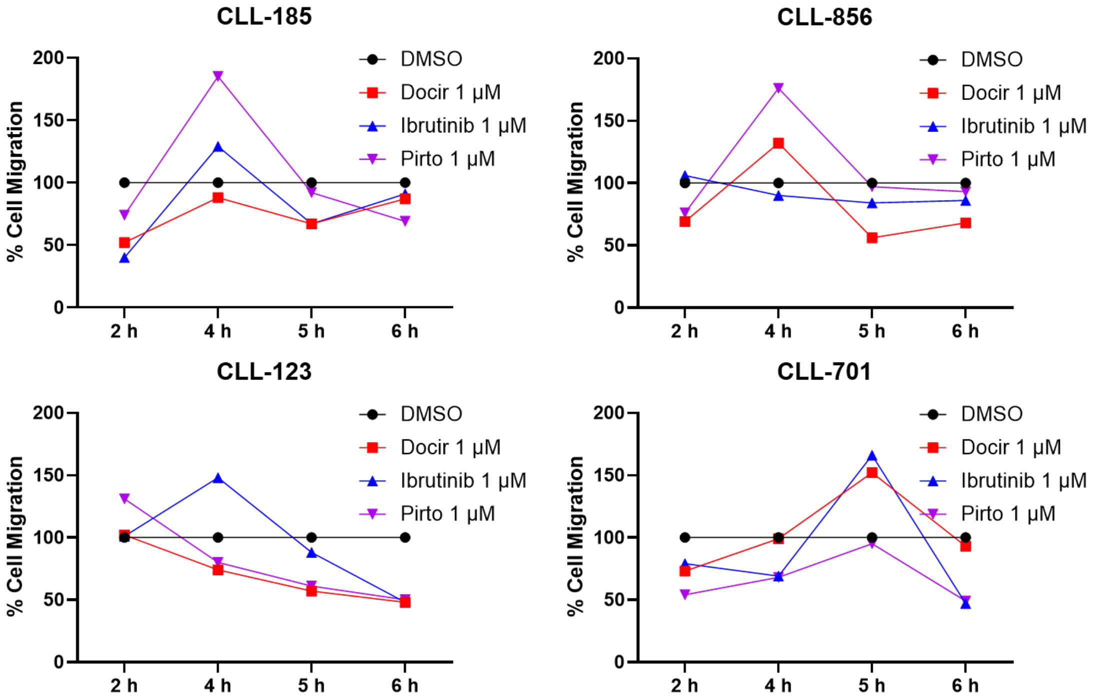

Supplemental Figure S1. Spontaneous migration dynamics during 6-hour incubation. Primary CLL cells were treated for 48 hours with BTKi, followed by migration analysis at 2,4,5,6 hours. Docir, docirbrutinib; Ibru, ibrutinib; ns, not significant; Pirto, pirtobrutinib

Supplemental Figure 2.

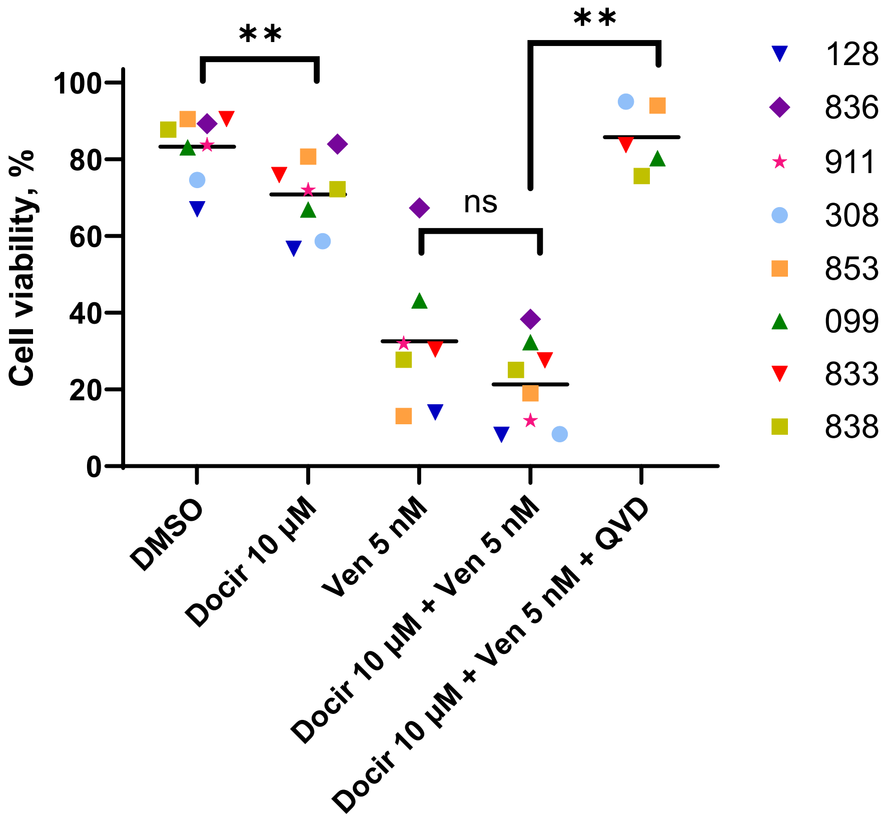

Supplemental Figure S2. Pan-caspase inhibition salvages docirbrutinib-induced apoptosis. Cells were treated with docirbrutinib, venetoclax and combination for 72 hours with BTK and annexin V/propidium iodide (PI)–negative events were analyzed. Ven, venetoclax; Docir, docirbrutinib; ns, not significant; * p<0.05, ** p<0.01, *** p<0.001, **** p<0.0001.

Supplemental Figure 3.

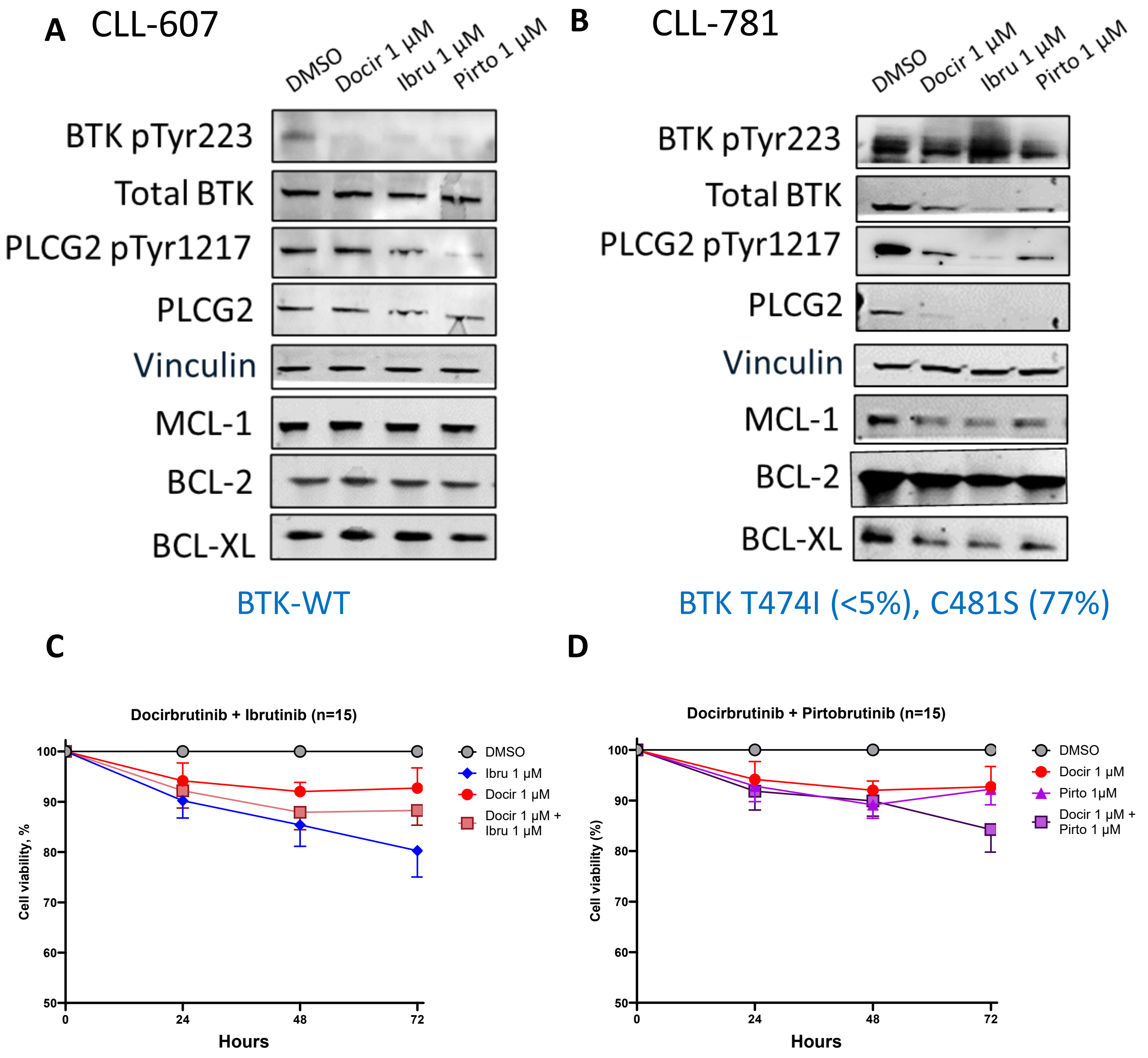

Supplemental Figure S3. Docirbrutinib inhibited BTK and PLCγ2 phosphorylation in pretreated CLL cells. A&B: BTK WT (CLL-607) and BTK-mutated (CLL-781) CLL cells were incubated with indicated BTKi and protein extracts were made and analyzed using immunoblot for phospho and total proteins. **C&D:** Cells were treated for 72 hours with BTKi and its combinations at equimolar concentrations, and annexin V/propidium iodide (PI)–negative events were analyzed. Docir, docirbrutinib; Ibru, ibrutinib; ns, not significant; Pirto, pirtobrutinib; WT, wild type

Supplemental Figure 4.

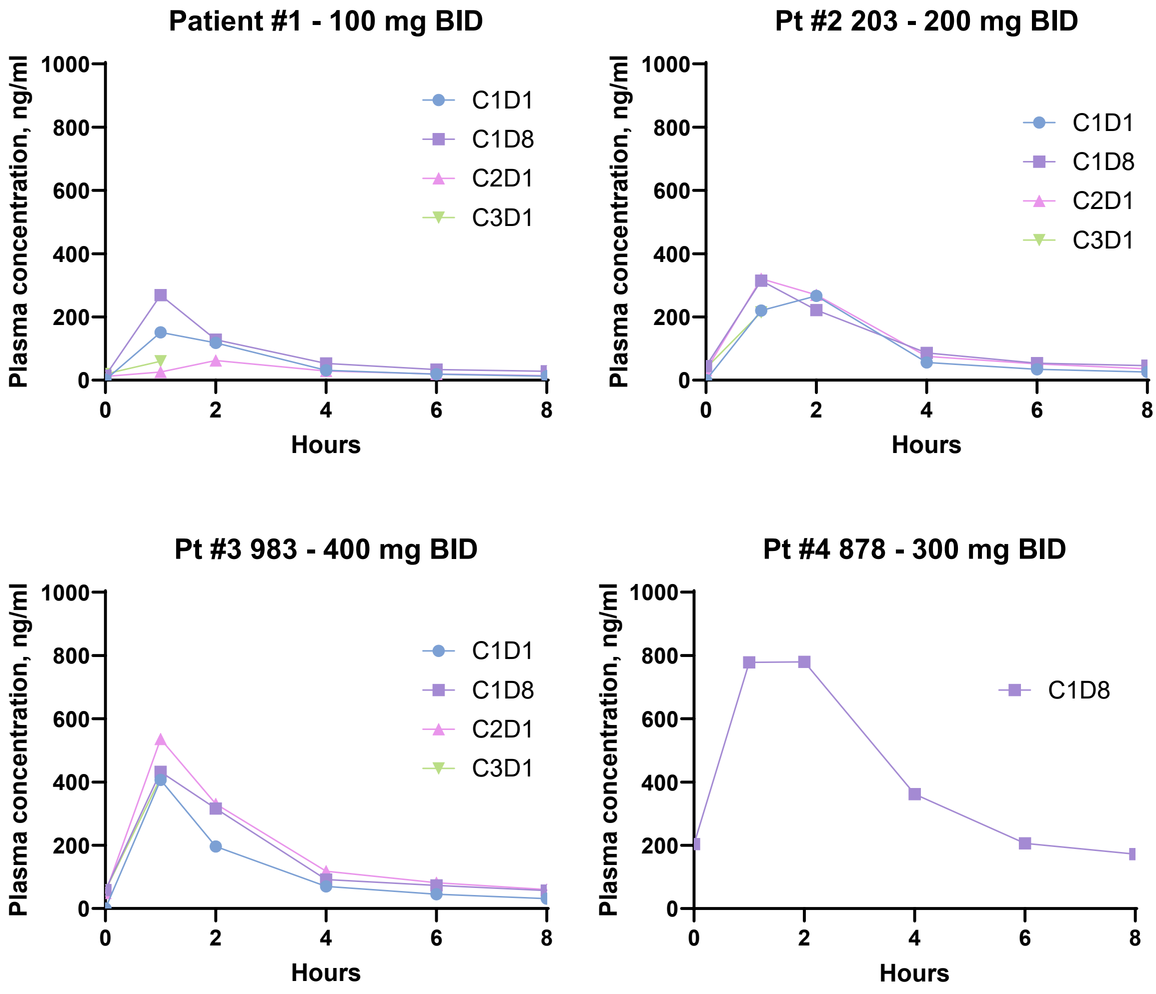

Supplemental Figure S4. Peak plasma level of docirbrutinib at various time points. Blood samples were collected at indicated times on C1D1, C1D8, C2D1 and C3D1, and plasma was analyzed for docirbrutinib levels. BID, twice a day; CxDx, cycle x day x;

Supplemental Figure 5.

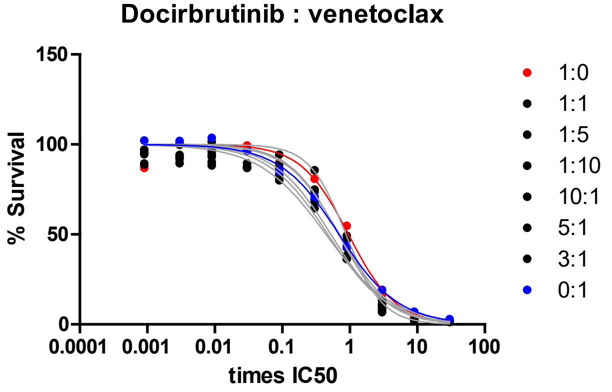

Docir : VEN	IC50 (times IC_{50})	Docir (nM)	VEN (nM)
1:0	0.96	1.72	0
1:1	0.47	0.43	1.52
1:5	0.44	0.13	2.37
1:10	0.68	0.11	3.96
10:1	0.88	1.44	0.51
5:1	0.70	1.05	0.75
3:1	0.52	0.71	0.84
0:1	0.70	0	4.45

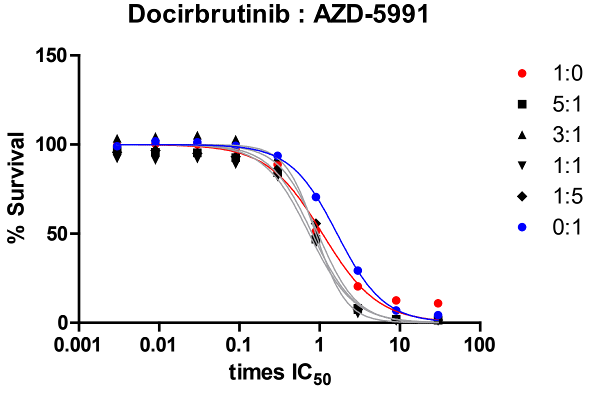

Docir : AZD	IC50 (times IC_{50})	Docir (nM)	AZD (nM)
1:0	1.09	7.24	0
5:1	0.81	4.51	11.70
3:1	0.90	4.49	19.42
1:1	0.74	2.47	32.03
1:5	0.95	1.06	68.86
0:1	1.65	0	142.96

Supplemental Figure S5. Dose-response curves of various docirbrutinib: venetoclax/AZD5991 ratios. IC_{50} values of docirbrutinib and venetoclax **(top)** and docirbrutinib and AZD5991 **(bottom)** combinations in OCI-Ly10. Cell viability was determined by resazurin assay at 96 hours. AZD, AZD5991; Docir, docirbrutinib; IC_{50}, half maximal inhibitory concentration; Ven, venetoclax.

Supplemental Figure 6.

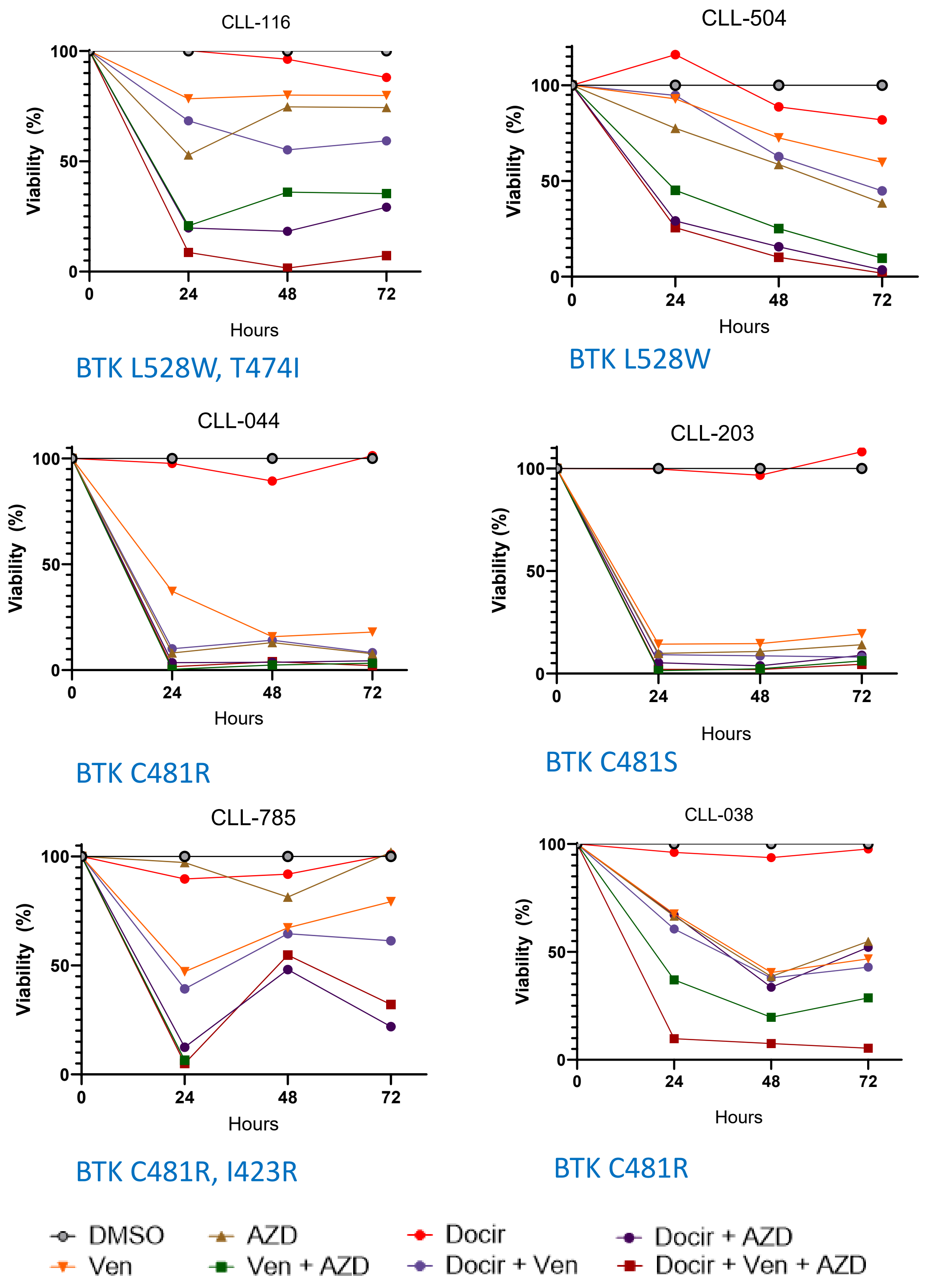

Supplemental Figure S6. Pharmacological profiling of 6 CLL patients. Cells BTK mutations were treated with docirbrutinib, venetoclax, AZD5991 and combination for 72 hours with BTK and annexin V/propidium iodide (PI)–negative events were analyzed every 24 hours. AZD, AZD5991; Docir, docirbrutinib; Ven, venetoclax.
